# Assessment of Reported Comparative Effectiveness and Safety of Atypical Antipsychotics in the Treatment of Behavioral and Psychological Symptoms of Dementia: A Network Meta-analysis

**DOI:** 10.1001/jamanetworkopen.2019.0828

**Published:** 2019-03-22

**Authors:** Ismaeel Yunusa, Adnan Alsumali, Asabe E. Garba, Quentin R. Regestein, Tewodros Eguale

**Affiliations:** 1 School of Pharmacy, Massachusetts College of Pharmacy and Health Sciences, Boston; 2 College for Public Health and Social Justice, Saint Louis University, St Louis, Missouri; 3 Department of Psychiatry, Brigham and Women's Hospital, Harvard Medical School, Boston, Massachusetts; 4 Division of General Internal Medicine, Brigham and Women's Hospital, Boston, Massachusetts

## Abstract

**Importance:**

Atypical antipsychotics offer modest effectiveness compared with placebo but with serious safety risks, including a boxed warning for the risk of death in the treatment of behavioral and psychological symptoms of dementia (BPSD). Their comparative effectiveness and safety are not fully known.

**Objective:**

To assess the relative benefits and safety of atypical antipsychotics in the treatment of BPSD shown in randomized clinical trials using network meta-analysis.

**Data Sources:**

PubMed/MEDLINE, Embase, PsychINFO, and Cochrane Library were searched from their inception until May 31, 2018. Key terms included dementia and atypical antipsychotics.

**Study Selection:**

Randomized clinical trials comparing any atypical antipsychotic with another atypical antipsychotic or with placebo were included in the analysis.

**Data Extraction and Synthesis:**

Two independent reviewers used a standardized data extraction and quality assessment form. Random-effects network meta-analyses were performed. Effect sizes were reported as standardized mean differences (SMDs) for continuous outcomes and odds ratios (ORs) for dichotomous outcomes with 95% CIs. In addition to ORs, the surface under the cumulative ranking curve (SUCRA) was ascertained, which represents the percentage of the effectiveness or safety for each treatment compared with a hypothetical treatment that would be ranked first without uncertainty.

**Main Outcomes and Measures:**

The primary effectiveness outcome assessed was the Neuropsychiatric Inventory (NPI); secondary effectiveness outcomes were the Brief Psychiatric Rating Scale (BPRS) and Cohen-Mansfield Agitation Inventory (CMAI). The primary safety outcomes were death and cerebrovascular adverse events (CVAEs). Secondary safety outcomes were extrapyramidal signs/symptoms; somnolence/sedation; falls, fracture, or injury; and urinary tract infection/incontinence.

**Results:**

Seventeen studies (5373 patients) were included. The mean (SD) age of all participants was 80.8 (3.1) years, and most were women (3748 [69.8%]). Compared with placebo, aripiprazole was associated with improvement in outcomes on the NPI (SMD, −0.17; 95% CI, −0.31 to −0.02), BPRS (SMD, −0.20; 95% CI, −0.35 to −0.05), and CMAI (SMD, −0.30; 95% CI, −0.55 to −0.05); quetiapine was associated with improvement in outcomes on the BPRS (SMD, −0.24; 95% CI, −0.46 to −0.01), and risperidone was associated with improvement in outcomes on the CMAI (SMD, −0.26; 95% CI, −0.37 to −0.15). Differences between atypical antipsychotics were not significant for effectiveness, death, or CVAE. Compared with placebo, risperidone (OR, 3.85; 95% CI, 1.55-9.55) and olanzapine (OR, 4.28; 95% CI, 1.26-14.56) were associated with increased risk of CVAEs. The SUCRA estimated relative ranking of treatments suggested that aripiprazole might be the most effective and safe atypical antipsychotic and that olanzapine provides the least benefit overall; however, these results should be interpreted with caution where point estimates (OR and SMD) show that there is no statistically significant difference.

**Conclusions and Relevance:**

This network meta-analysis supports the existence of a trade-off between the effectiveness and safety of atypical antipsychotics in the treatment of BPSD and confirms that a single most effective and safe treatment option does not exist. Clinicians should individualize the assessment of safety risks against expected benefits when prescribing these medications to patients with dementia.

## Key Points

**Question:**

Which atypical antipsychotic is most beneficial and safe for the treatment of behavioral and psychological symptoms of dementia?

**Findings:**

In this network meta-analysis of 17 studies (5373 patients), no significant differences were found across measures of effectiveness and safety among aripiprazole, olanzapine, quetiapine, and risperidone, although differences were found for some of these drugs and outcomes compared with placebo. No trials were found for other atypical antipsychotics.

**Meaning:**

Insufficient evidence exists on which atypical antipsychotic is both safest and most beneficial across several measures for people with dementia, and this study suggests that a single most effective and safe treatment option may not exist.

## Teaser

This network meta-analysis examines the results of randomized clinical trials that evaluated the effectiveness and safety of atypical antipsychotics in patients with dementia.

## Introduction

Dementia is a progressive, degenerative disorder characterized by cognitive and functional impairment. The behavioral and psychological symptoms of dementia (BPSD), otherwise known as the neuropsychiatric symptoms of dementia, represent a spectrum of noncognitive disturbances.^1^ These disturbances can include aggression, agitation, delusion, hallucination, anxiety, psychosis, depression, sleep or appetite changes, and apathy.^2^ More than 90% of patients with dementia develop at least 1 of these symptoms during the course of their disease.^3,4^ Behavioral and psychological symptoms of dementia decrease the quality of life of patients with dementia and increase their chances of being institutionalized.^5^

Atypical antipsychotics (AAPs) include aripiprazole, olanzapine, quetiapine, and risperidone. Risperidone is licensed for the treatment of severe BPSD in Australia, Canada, New Zealand, and the United Kingdom but not in the United States. No other AAP has approval for this indication, so they are considered off-label when prescribed for BPSD. Evidence from pairwise meta-analyses of randomized clinical trials suggests that AAPs offer only modest improvement in BPSD but can cause serious adverse events, notably death and cerebrovascular adverse events (CVAEs).^6^ Therefore, the use of AAPs might seem unjustifiable given that their off-label use lacks strong scientific evidence and is generally associated with adverse events.^7,8^ Concerns about safety risks from AAPs began in 2002^9^ and there have been regulatory warnings since then from the European Medicines Agency, US Food and Drug Administration, and Health Canada.^10,11^ Despite these warnings, AAPs are used in about 12.3% to 37.5% of patients for the treatment of BPSD.^12^

In 2015, the American Geriatrics Society updated the Beers Criteria for potentially inappropriate medication use in older adults and recommended the avoidance of antipsychotics for BPSD unless nonpharmacologic options have failed or are not possible and the patient is threatening substantial harm to self or others.^13^ Its European variant, the Screening Tool of Older Person's Prescriptions (STOPP) and Screening Tool to Alert Doctors to the Right Treatment (START) also recommended avoiding antipsychotics for BPSD unless symptoms are severe and other nonpharmacologic treatments have failed.^14^

Previous pairwise meta-analyses have not determined which antipsychotic is most beneficial and safe for patients with BPSD as most trials compared AAPs with placebo and few with other AAPs. Toward this goal, the network meta-analysis (NMA), an extension of the traditional pairwise meta-analysis that enables simultaneous comparison of multiple interventions, can be helpful. The NMA generates evidence from direct and indirect comparisons within a network of trials and enables inference about every possible comparison between a pair of interventions in this network, even when some comparisons have never been directly evaluated in a trial.^15^

To our knowledge, no NMA has been performed comparing AAPs for the treatment of BPSD. Therefore, we aimed to use NMA to evaluate the results of randomized placebo-controlled or head-to-head trials on BPSD designed to determine the effectiveness and safety of different AAPs.

## Methods

### Literature Review

We conducted this study according to the Preferred Reporting Items for Systematic Reviews and Meta-analyses (PRISMA) extension statement for health care interventions.^16,17^ We searched the literature using the Cochrane Library, Embase, MEDLINE/PubMed, and PsychINFO databases from their inception to May 31, 2018, for studies evaluating the effectiveness and safety of AAPS for the treatment of BPSD. Key search terms included dementia, atypical antipsychotics, aripiprazole, olanzapine, risperidone, quetiapine, asenapine, clozapine, iloperidone, lurasidone, paliperidone, and ziprasidone. The full search strategy is described in eTable 1 in the Supplement. Reference lists of selected articles were examined to ensure that all relevant articles published through May 2018 were identified. All titles and abstracts were independently screened by 2 of us (I.Y., A.A.), and potentially relevant articles were selected for full-text review. This full-text screening was conducted independently by the same 2 reviewers, and any disagreements were resolved by consultation with a third reviewer (T.E.).

### Study Selection

Only randomized clinical trials comparing identified AAPs with placebo or head-to-head comparisons of different AAPS in adults 65 years or older with BPSD were included. We evaluated trials that compared at least 2 of the following AAPs with each other: aripiprazole, olanzapine, quetiapine, and risperidone. Trials that compared 1 of those AAPs with placebo were also included. Exclusion criteria were study designs other than randomized clinical trials, active-controlled trials comparing AAPs with any other medication, studies with less than 6 weeks of follow-up, and non-English articles.

### Data Extraction and Outcome Measures

Two of us (I.Y., A.A.) independently extracted the data according to an a priori standardized data extraction sheet. The primary outcome of effectiveness was an improvement in the Neuropsychiatric Inventory (NPI)^18,19^ total score. Changes in the Brief Psychiatric Rating Scale (BPRS)^20^ total score and the Cohen-Mansfield Agitation Inventory (CMAI)^21^ total score were secondary effectiveness outcomes. For safety outcomes, we evaluated overall mortality; CVAEs; falls, fracture, or injury; somnolence or sedation; extrapyramidal symptoms (EPSs); and urinary incontinence or urinary tract infection.

We also extracted information on the following characteristics: age, sex, setting (eg, nursing home, long-term care, or outpatient), site (eg, single or multiple), blinding, type of dementia (eg, Alzheimer disease, vascular, Lewy body, or mixed dementia), psychological symptoms, and baseline Mini-Mental State Examination score.

When necessary, we contacted authors to provide unpublished data. We did not consider eligible conference abstracts with no full text that were included in previous pairwise meta-analyses as they do not contain the information required to fully assess the distribution of potential effect modifiers necessary to make transitivity assumptions and to assess the risk of bias.

### Risk of Bias Assessment

Risk of bias was assessed independently by 2 of us (I.Y., A.A.) using the Cochrane Risk of Bias Tool.^22^ Each study was classified as having low, medium, or high risk of bias.

### Assessment of Clinical Assumptions

Transitivity (ie, distribution of patient and study characteristics that are potential modifiers of treatment effects and are sufficiently similar in different sets of trials before an indirect comparison) is a fundamental assumption underlying NMA.^23^ Thus, we evaluated the credibility of transitivity in our data by qualitatively assessing the distribution of the potential effect modifiers across the different direct comparisons.^24^

### Data Synthesis and Evaluation of Statistical Assumptions

The NMA for each outcome was performed using a multivariate meta-analysis approach with the network package in Stata Statistical Software, version 15.1 (StataCorp).^25,26^ Relative odds ratios (ORs) with 95% CIs for dichotomous outcomes and standardized mean differences (SMDs) for continuous outcomes are presented in the results for each possible network comparison. For clinical interpretation, SMD was re-expressed by using the Cohen rule of thumb for effect size (eg, <0.40, small; 0.40-0.70, moderate; and >0.70, large).^27^ The restricted maximum likelihood estimation method was used to estimate heterogeneity, assuming a common estimate for heterogeneity variance among different comparisons for each outcome.^28,29^ The predictive intervals of the network were estimated to evaluate the additional uncertainty expected in future studies owing to heterogeneity.^30^ Consistency was evaluated by examining the agreement between direct and indirect treatment effects in all closed loops and by assuming loop-specific heterogeneity using the loop-specific approach.^31,32,33^ To evaluate the presence of inconsistency for any treatment contrast in the network, the node-splitting analysis method was used because it assesses whether the direct and indirect evidence on a specific node are in agreement.^34,35^

In addition to estimation of ORs, treatment ranking was ascertained using the surface under the cumulative ranking curve (SUCRA), which represents the percentage of the effectiveness or safety for each treatment compared with a hypothetical treatment that would be ranked first without uncertainty.^28,36^ Hierarchical cluster ranking based on SUCRAs for pairs of outcomes was used to show the relative effectiveness and safety of the drugs and placebo on a graph in which the upper right quadrant represents the more effective and more safe values, the lower right quadrant represents the more effective but less safe, the lower left quadrant represents the less effective and less safe, and the upper left quadrant represents the less effective and more safe values.

### Small-Study Effects and Additional Analyses

To assess whether small studies tended to give different effectiveness or safety results, comparison-adjusted funnel plots were evaluated for each primary outcome.^37^ We focused on comparisons of all active interventions against placebo that might be prone to small-study effects. We then conducted sensitivity analyses for the primary outcomes in which we excluded studies with a sample size of 100 or less^38^ to assess the robustness of our findings.

## Results

### Characteristics and Risk of Bias of the Included Studies

**Figure 1.**
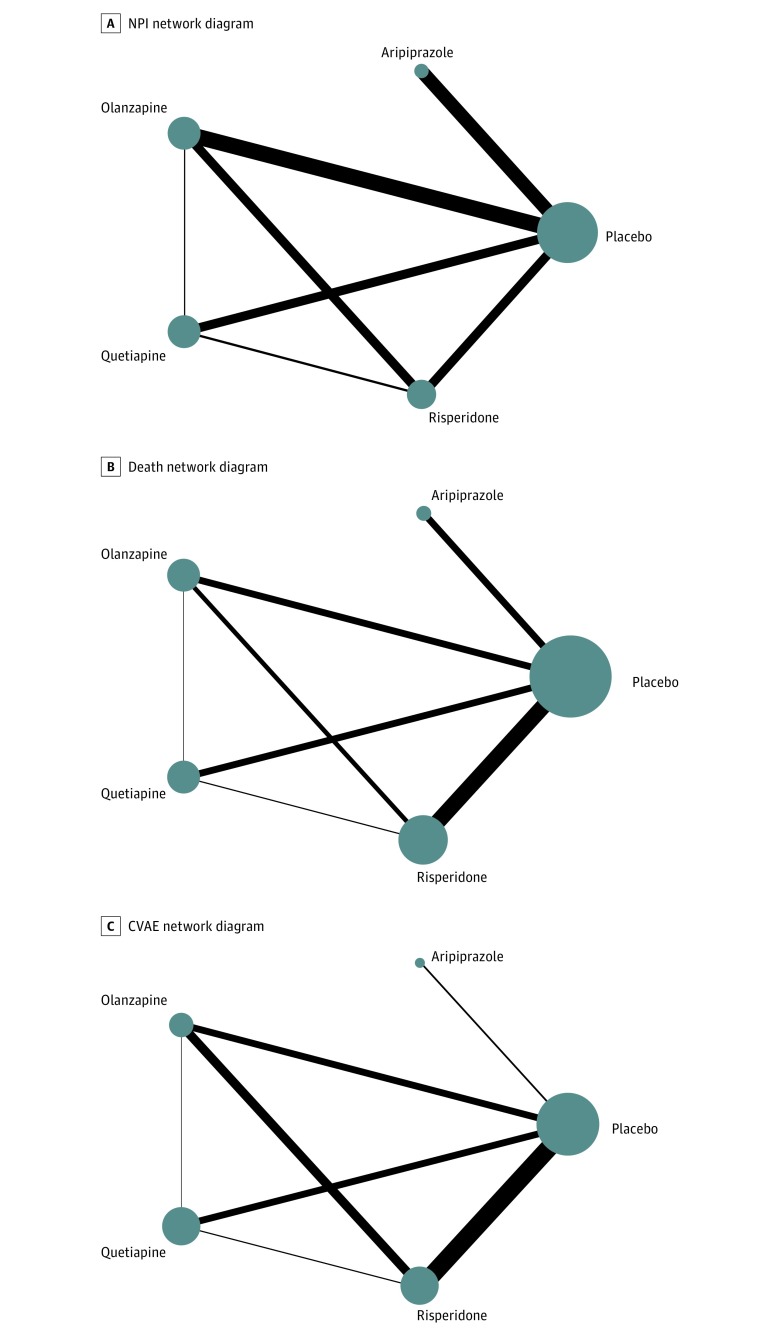
Network Diagrams A, Neuropsychiatric Inventory (NPI). B, Death. C, Cerebrovascular adverse events (CVAE). Network diagrams showing how atypical antipsychotics were compared in clinical trials with respect to number of studies and sample sizes. The width of the lines is proportional to the number of trials directly comparing every pair of treatments, and the size of every node is proportional to the number of randomized participants (sample size).

From the literature search, a total of 17 clinical trials^39,40,41,42,43,44,45,46,47,48,49,50,51,52,53,54,55^ with 5373 participants were included for analysis. The study selection process is illustrated in a PRISMA flowchart (eFigure 1 in the Supplement). The median duration of follow-up was 10 weeks (range, 6-32 weeks). Twelve studies were conducted in nursing homes, 3 in outpatient settings, and 2 in both nursing homes and outpatient settings (eTable 2 in the Supplement). The risk of bias assessment is presented in eTable 3 in the Supplement. The network plots for eligible comparisons for the primary outcomes are shown in Figure 1, and those for the secondary outcomes are presented in eFigure 2 in the Supplement. Quetiapine and risperidone were involved in most of the comparisons (7 of 17 trials), followed by olanzapine (4) and aripiprazole (3); no eligible trials were available for asenapine, clozapine, iloperidone, lurasidone, paliperidone, and ziprasidone.

### Evaluation of Clinical Assumptions

The mean (SD) age of all participants was 80.8 (3.1) years, and most were women (3748 [69.8%]). All patients had a diagnosis of BPSD, and some had diagnoses of Alzheimer disease, vascular, Lewy body, or mixed dementia. The distribution with regard to age, sex, and BPSD diagnosis was comparable between studies (eTable 2 in the Supplement). Thus, the transitivity assumption is tenable in our data set.

### Effectiveness Outcomes 

#### NPI Results

The NMA suggested that aripiprazole (SMD, −0.17; 95% CI, −0.31 to −0.02) was associated with improvement on the NPI compared with placebo, while olanzapine, quetiapine, and risperidone were not. There was no statistically significant difference between included AAPs on the NPI (Table 1). According to the SUCRA, aripiprazole (85.3%) had the highest probability of effectiveness on the NPI (eFigure 3A in the Supplement).

**Table 1.** League Table Comparing Atypical Antipsychotics With Respect to the Neuropsychiatric Inventory^a^

Treatment	Quetiapine	Olanzapine	Aripiprazole	Placebo
Risperidone	0.07 (−0.15 to 0.30)	0.10 (−0.08 to 0.27)	0.16 (−0.07 to 0.40)	-0.01 (−0.19 to 0.18)
Quetiapine		0.02 (−0.18 to 0.23)	0.09 (−0.14 to 0.31)	−0.08 (−0.25 to 0.09)
Olanzapine			0.06 (−0.13 to 0.26)	−0.10 (−0.24 to 0.03)
Aripiprazole				−0.17 (−0.31 to −0.02)^b^

^a^ Comparisons are based on standardized mean differences between treatments. Comparisons should be read from left to right, and the estimate is in the cell shared between the column-defining treatment and the row-defining treatment.

^b^ Significant results (95% CI for standardized mean differences does not include 0).

#### BPRS Results

Aripiprazole (SMD, −0.20; 95% CI, −0.35 to −0.05) and quetiapine (SMD, −0.24; 95% CI, −0.46 to −0.01) were associated with improvement on the BPRS compared with placebo; olanzapine and risperidone were not (eFigure 4A in the Supplement). No statistically significant differences were found between included AAPs on the BPRS. According to the SUCRA, quetiapine (80.2%) and aripiprazole (72.9%) had the highest probability of effectiveness on the BPRS (eFigure 3B in the Supplement).

#### CMAI Results 

Aripiprazole (SMD, −0.30; 95% CI, −0.55 to −0.05) and risperidone (SMD, −0.26; 95% CI, −0.37 to −0.15) were associated with improvement on the CMAI compared with placebo; olanzapine and quetiapine were not (eFigure 4B in the Supplement). There was no statistically significant difference between included AAPs on the CMAI. According to the SUCRA, aripiprazole (73.8%) and risperidone (68.6%) had the highest probability of effectiveness on the CMAI (eFigure 3C in the Supplement).

### Safety Outcomes

#### Mortality

The NMA suggested that none of the included AAPs were significantly different from placebo or from each other on the risk of death (Table 2), although 95% CIs were wide owing to small numbers of events (eFigure 4C in the Supplement). According to the SUCRA, placebo had the highest probability of safety on the mortality outcome (87.3%), followed by risperidone (55.4%), aripiprazole (37.9%), quetiapine (37.1%), and olanzapine (32.4%) (eFigure 3D in the Supplement).

**Table 2.** League Table Comparing Atypical Antipsychotics With Respect to Death Outcome^a^

Treatment	Quetiapine	Olanzapine	Aripiprazole	Placebo
Risperidone	0.81 (0.32-2.02)	0.76 (0.31-1.84)	0.80 (0.27-2.36)	1.32 (0.77-2.27)
Quetiapine		0.94 (0.31-2.88)	0.99 (0.29-3.39)	1.64 (0.74-3.63)
Olanzapine			1.05 (0.30-3.73)	1.74 (0.74-4.07)
Aripiprazole				1.66 (0.65-4.25)

^a^ Comparisons are based on odds ratios (95% CIs) between treatments. Comparisons should be read from left to right, and the estimate is in the cell shared between the column-defining treatment and the row-defining treatment.

#### Cerebrovascular Adverse Events

Compared with placebo, olanzapine (OR, 4.28; 95% CI, 1.26-14.56) and risperidone (OR, 3.85; 95% CI, 1.55-9.55) were associated with a significantly increased risk of CVAEs; aripiprazole (OR, 1.09; 95% CI, 0.12-9.46) and quetiapine (OR, 1.36; 95% CI, 0.43-4.25) were not (Table 3). None of the included AAPs were significantly different from each other on the risk of CVAEs (eFigure 4D in the Supplement).

**Table 3.** League Table Comparing Atypical Antipsychotics With Respect to Cerebrovascular Adverse Events^a^

Treatment	Quetiapine	Olanzapine	Aripiprazole	Placebo
Risperidone	2.84 (0.76-10.59)	0.90 (0.32-2.56)	3.54 (0.34-37.03)	3.85 (1.55-9.55)^b^
Quetiapine		0.32 (0.07-1.42)	1.25 (0.11-14.42)	1.36 (0.43-4.25)
Olanzapine			3.94 (0.33-47.33)	4.28 (1.26-14.56)^b^
Aripiprazole				1.09 (0.12-9.46)

^a^ Comparisons are based on odds ratios (95% CIs) between treatments. Comparisons should be read from left to right, and the estimate is in the cell shared between the column-defining treatment and the row-defining treatment.

^b^ Significant results (95% CI for odds ratio does not include 1).

According to the SUCRA, placebo had the highest probability of safety on the CVAE outcome (80.4%), followed by aripiprazole (69.1%), quetiapine (65.1%), risperidone (19.6%), and olanzapine (15.8%) (eFigure 3E in the Supplement).

#### Extrapyramidal Signs and Symptoms

Compared with placebo, risperidone was associated with a significantly increased risk of EPSs (OR, 2.23; 95% CI, 1.56-3.18), while aripiprazole (OR, 1.26; 95% CI, 0.53-2.97), olanzapine (OR, 1.54; 95% CI, 0.97-2.46), and quetiapine (OR, 0.59; 95% CI, 0.27-1.33) were not (eFigure 4E in the Supplement).

Risperidone was associated with an increased risk of EPSs compared with quetiapine (OR, 3.75; 95% CI, 1.61-8.73). Quetiapine was associated with a decreased risk of EPSs compared with olanzapine (OR, 0.39; 95% CI, 0.16-0.93). The other AAPs were not significantly different from each other. According to the SUCRAs for EPSs, quetiapine (94.2%) and aripiprazole (48.8%) ranked as the safest agents, followed by olanzapine (34.0%); risperidone (3.7%) ranked as the worst (eFigure 3F in the Supplement).

#### Somnolence or Sedation

Compared with placebo, all the included AAPs were associated with a significantly increased risk of somnolence or sedation (eFigure 4F in the Supplement). Risperidone was associated with a decreased risk of somnolence or sedation compared with olanzapine (OR, 0.63; 95% CI, 0.41-0.96) or quetiapine (OR, 0.58; 95% CI, 0.34-0.97). The other AAPs were not significantly different from each other.

According to the SUCRAs for somnolence or sedation, placebo (99.9%) was ranked as the safest, followed by risperidone (66.2%), aripiprazole (45.4%), and olanzapine (23.0%). Quetiapine (15.5%) was the least safe (eFigure 3G in the Supplement).

#### Falls, Fracture, or Injury

Compared with placebo, risperidone (OR, 0.79; 95% CI, 0.64-0.98) was associated with a decreased risk of falls, fracture, or injury, while the other AAPs were not (eFigure 4G in the Supplement). Risperidone (OR, 0.63; 95% CI, 0.43-0.94) was associated with decreased risk of falls, fracture, or injury compared with olanzapine. The other AAPs were not significantly different from each other.

According to the SUCRAs for falls, fracture, or injury, risperidone (81.6%) and quetiapine (79.5%) ranked in the top 2 positions for safety, followed by aripiprazole (43.8%), placebo (36.4%), and olanzapine (8.8%) (eFigure 3H in the Supplement).

#### Urinary Incontinence or Urinary Tract Infection

Compared with placebo, quetiapine (OR, 2.11; 95% CI, 1.05-4.26) was associated with increased urinary incontinence or urinary tract infection; the other AAPs were not (eFigure 4H in the Supplement). The other AAPs were not significantly different from each other.

According to the SUCRA for urinary incontinence or urinary tract infection, placebo had the highest probability of safety (85.8%), followed by olanzapine (66.9%), risperidone (46.6%), aripiprazole (36.0%), and quetiapine (14.6%) (eFigure 3I in the Supplement).

### Simultaneous Ranking of the Interventions

**Figure 2.**
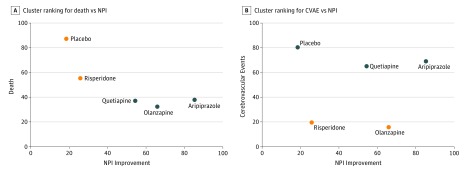
Cluster Ranking Plot for Relative Effectiveness and Safety A, Cluster ranking for death vs Neuropsychiatric Inventory (NPI). B, Cluster ranking for cerebrovascular adverse events (CVAEs) vs NPI. The plot is based on cluster analysis of surface under the cumulative ranking curve (SUCRA) values. Each plot shows SUCRA values on a scale of 0% to 100% for 2 outcomes. Each color represents a group of treatments that belongs to the same cluster. The upper right quadrant represents the more effective and more safe treatments; lower right quadrant, more effective but less safe; lower left quadrant, less effective and less safe; and upper left quadrant, less effective and more safe.

Cluster ranking for NPI (effectiveness) vs death (safety) outcomes showed that placebo was least effective and most safe, with risperidone falling into the same (upper left quadrant; less effective/more safe) cluster as placebo. Aripiprazole was the most effective, with olanzapine and quetiapine falling into the same (lower right quadrant; more effective/less safe) cluster as aripiprazole (Figure 2A).

Cluster ranking for NPI (effectiveness) vs CVAE (safety) outcomes (Figure 2B) again showed that placebo was least effective and most safe. In this analysis, aripiprazole and quetiapine formed a more effective/more safe cluster, while risperidone and olanzapine formed a cluster with very low safety.

### Evaluation of Statistical Inconsistency

The loop-specific approach did not suggest any inconsistency between closed loops (eTable 4 in the Supplement). Similarly, side-splitting did not suggest the presence of statistical inconsistency for any outcome (eTable 5 in the Supplement).

### Small-Study Effects and Additional Analyses

Comparison-adjusted funnel plots indicated evidence of a publication bias or small-study effects for NPI, mortality, and CVAE outcomes (eFigure 5 in the Supplement). Sensitivity analyses that excluded studies with small sample sizes gave similar but less precise results compared with the primary analyses (eFigure 6 in the Supplement).

## Discussion

This NMA found that no AAP was consistently associated with better results than the others across all effectiveness and safety outcomes. However, aripiprazole was associated with improvement on NPI, BPRS, and CMAI outcomes compared with placebo. Quetiapine was associated with improvement on the BPRS compared with placebo but not on the CMAI or NPI, while risperidone was associated with improvement on the CMAI compared with placebo but not in the BPRS and NPI. Olanzapine was not associated with better results than placebo on any of the effectiveness outcomes. The effect size of aripiprazole, risperidone, and quetiapine over placebo was small and consistent with previous findings.^56,57,58,59^

None of the AAPs was associated with greater safety profile than another regarding the risk of death. Previous pairwise meta-analyses pooled all AAPs and found an increased risk of death vs placebo^60,61,62,63,64^; however, because we focused on the individual drugs, the results are not comparative. Most of the results from the SUCRA ranking are congruent with published observational studies. For example, previous reports corroborate ranking quetiapine as the safest and risperidone as the least safe based on EPSs.^65,66^ Quetiapine was ranked as most likely to cause somnolence or sedation, which might explain why it is commonly used as a sedative-hypnotic in patients with dementia.^67^ The ranking results for somnolence or sedation are similar to those of death and may be explained by the fact that excessive sedation or sleep is associated with infection, including pneumonia, which could ultimately lead to death.^68,69^ There is no evidence to support or justify the use of asenapine, clozapine, iloperidone, lurasidone, paliperidone, and ziprasidone given the lack of studies conducted on their use.

This study found risperidone and olanzapine to be associated with an increased risk of CVAEs compared with placebo. Although the 3-fold increase in CVAE risk from the use of risperidone corroborates evidence from previous pairwise meta-analyses,^70^ the increase associated with olanzapine represents the first significant association from a meta-analysis. Previous meta-analyses revealed olanzapine has a higher incidence of CVAEs than placebo, but not a statistically significant association as revealed by this NMA.^71,72^ Whenever physicians consider prescribing risperidone and olanzapine, risk factors for CVAEs should be assessed. Our findings support the American Psychiatric Association guidelines and STOPP/START criteria, which recommend that olanzapine and risperidone should be avoided or used with caution in individuals with hypovolemia, history of cerebrovascular and cardiac diseases, and vascular dementia who are already at high risk for stroke or transient ischemic attack.

We found that all included AAPs were associated with increased risk of somnolence or sedation compared with placebo, although risperidone was associated with less risk of somnolence or sedation than olanzapine and quetiapine. This finding implies that AAPs should be avoided when they are sedating a patient. The use of quetiapine was associated with a reduced risk of EPSs in comparison with olanzapine and risperidone; risperidone was associated with an increased risk compared with placebo. Patients with BPSD and either Lewy body dementia or Parkinson disease should avoid the use of risperidone to prevent worsening of EPSs. The ranking of quetiapine and aripiprazole as the safest for avoiding EPSs supports their exclusion from the American Geriatrics Society 2015 Beers Criteria recommendation for avoiding antipsychotics in elderly patients with dementia; these 2 drugs have the lowest propensity to precipitate worsening of Parkinson disease. In contrast to a large observational study of 195 554 individuals that did not find a difference among AAPs for the risk of fracture and falls,^73^ we found that risperidone was associated with reduced risk of injury, fracture, or falls compared with olanzapine. The association of risperidone’s greater safety profile than olanzapine in our study was in line with a published systematic review.^74^

Our NMA supports the existence of a trade-off between the effectiveness and safety of AAPs in the treatment of BPSD and confirms that a single most effective and safe treatment option does not exist. Until new effective therapies are developed, the use of all AAPs for BPSD remains controversial because they offer only a modest benefit over placebo but confer considerable safety risks. The American Psychiatric Association Practice Guideline recommends that, before treatment of BPSD with an antipsychotic is initiated, the potential risks and benefits should be evaluated by the clinician and discussed with the patient and their proxy decision maker or family.^8^ Treatment with antipsychotics should be well documented in a patient's medical record and carefully monitored. While most of the effect estimates were not statistically significant, the cluster ranking graph illustrating the relative safety and effectiveness might be considered as a guide in treating BPSD until further evidence is generated or new treatments are developed.

In general, when considering prescribing AAPs to patients with BPSD, safety risks can be minimized by encouraging family caregivers to implement the structured DICE (describe, investigate, create, and evaluate) care approach first. DICE methodically guides health care clinicians on how to carefully screen patients to identify those who might benefit from antipsychotic medications and those whose condition might worsen.^75^

### Limitations

This NMA was limited to 17 eligible studies. The inclusion of additional studies would have provided more precise outcome estimations. Insufficient data from the eligible studies prevented the exploration of possible causes of death, such as pneumonia and cardiac-related adverse events. Individual patient data meta-analyses could identify these characteristics, and future studies will include patient-level data from the clinical trials’ databases through the sponsors or authors.

## Conclusions

This study supports the existence of a trade-off between the effectiveness and safety of AAPs in the treatment of BPSD and confirms that a single most effective and safe treatment option does not exist. Clinicians should individualize the assessment of safety risks against expected benefits when prescribing these medications to patients with dementia. Future studies are needed that include individual patient data in the NMA toward identifying specific individual characteristics that may influence the effectiveness or safety of AAPs.

## Notes

Supplement. **eTable 1.** Search Strategy **eTable 2.** Study and Patient Characteristics **eTable 3.** Risk of Bias Assessment **eTable 4.** Inconsistency Test Based on Loop-Specific Approach **eTable 5.** Inconsistency Test Based on Side-Splitting Approach **eFigure 1.** PRISMA Study Selection Flowchart **eFigure 2.** Network Plots for Secondary Outcomes **eFigure 3**. Surface Under the Cumulative Ranking Curves (SUCRAs) Showing the Percentage Of Effectiveness Against an Imaginary Treatment That Should Rank as the Most Effective Without Uncertainty **eFigure 4**. Interval Plots **eFigure 5**. Comparison-adjusted funnel plots for Primary Outcomes Involving All Studies Comparing All AAPs vs Placebo **eFigure 6**. Sensitivity Analysis Removing Studies With Small Sample Size **eReferences**
