# Supplementary material for: Assessment of Reported Comparative Effectiveness and Safety of Atypical Antipsychotics in the Treatment of Behavioral and Psychological Symptoms of Dementia: A Network Meta-analysis
Source: JAMA Netw Open. 2019 Mar 22;2(3):e190828. doi: 10.1001/jamanetworkopen.2019.0828 (PMC6583313; doi:10.1001/jamanetworkopen.2019.0828)
Supplement: Supplement. — eTable 1. Search Strategy eTable 2. Study and Patient Characteristics eTable 3. Risk of Bias Assessment eTable 4. Inconsistency Test Based on Loop-Specific Approach eTable 5. Inconsistency Test Based on Side-Splitting Approach eFigure 1. PRISMA Study Selection Flowchart eFigure 2. Network Plots for Secondary Outcomes eFigure 3. Surface Under the Cumulative Ranking Curves (SUCRAs) Showing the Percentage Of Effectiveness Against an Imaginary Treatment That Should Rank as the Most Effective Without Uncertainty eFigure 4. Interval Plots eFigure 5. Comparison-adjusted funnel plots for Primary Outcomes Involving All Studies Comparing All AAPs vs Placebo eFigure 6. Sensitivity Analysis Removing Studies With Small Sample Size eReferences [file jamanetwopen-2-e190828-s001.pdf]

Supplementary Online Content

Yunusa I, Alsumali A, Garba AE, Regestein QR, Eguale T. Assessment of reported comparative effectiveness and safety of atypical antipsychotics in the treatment of behavioral and psychological symptoms of dementia: a network meta-analysis. *JAMA Netw Open*. 2019;2(3):e190828. doi:10.1001/jamanetworkopen.2019.0828

eTable 1. Search Strategy

eTable 2. Study and Patient Characteristics

eTable 3. Risk of Bias Assessment

eTable 4. Inconsistency Test Based on Loop-Specific Approach

eTable 5. Inconsistency Test Based on Side-Splitting Approach

eFigure 1. PRISMA Study Selection Flowchart

eFigure 2. Network Plots for Secondary Outcomes

eFigure 3. Surface Under the Cumulative Ranking Curves (SUCRAs) Showing the Percentage of Effectiveness Against an Imaginary Treatment That Should Rank as the Most Effective Without Uncertainty

eFigure 4. Interval Plots

eFigure 5. Comparison-adjusted funnel plots for Primary Outcomes Involving All Studies Comparing All AAPs vs Placebo

eFigure 6. Sensitivity Analysis Removing Studies with Small Sample Size

eReferences

This supplementary material has been provided by the authors to give readers additional information about their work.

© 2019 Yunusa I et al. *JAMA Network Open.*

eTable 1: SEARCH STRATEGY

Database	Search Strategy	Number of hits (As of May 2018)
PubMed/MEDLINE	(("Dementia"[Mesh] or Dementia*[tw] or Amentia* [tw] or Senile Paranoid Dementia [tw] or Familial Dementia* [tw])<br><br>AND<br><br>("Quetiapine Fumarate"[Mesh] or Seroquel [tw] or Quetiapine [tw] or "Risperidone"[Mesh] or Risperdal Consta [tw] or Consta, Risperdal [tw] or Risperdal [tw] or ziprasidone [tw] or ziprasidone hydrochloride [tw] or ziprasidone hydrochloride, monohydrate [tw] or "Aripiprazole"[Mesh] OR Aripiprazole[tw] OR Abilify[tw] OR Asenapine[tw] OR Asenapine maleate[tw] OR saphris[tw] OR "Clozapine"[Mesh] OR Clozaril[tw] OR Leponex[tw] OR Iloperidone[tw] OR Fanapt[tw] OR "Lurasidone Hydrochloride"[Mesh] OR Lurasidone[tw] OR Lurasidone HCL[tw] OR Latuda[tw] OR olanzapine[tw] OR Symbyax[tw] OR Zyprexa[tw] OR "Paliperidone Palmitate"[Mesh] OR Paliperidone Palmitate[tw] OR Paliperidone[tw] OR Invega[tw] or "aripiprazole lauroxil" [Supplementary Concept] or "Asenapine" [Supplementary Concept] or "iloperidone" [Supplementary Concept] or "olanzapine" [Supplementary Concept] or "ziprasidone" [Supplementary Concept]))<br><br>AND<br><br>("Randomized Controlled Trial" [Publication Type] or "Randomized Controlled Trials as Topic"[Mesh] or Clinical Trials, randomized [tw] or Trials, Randomized Clinical [tw] or Controlled Clinical Trials, Randomized [tw] or Randomized Controlled Trial [tw])	145
Embase	(aripiprazole/ or aripiprazole monohydrate.tw. or abilify.tw. or asenapine/ or asenapine.tw. or asenapine maleate.tw. or saphris.tw. or sycrest.tw. or alemoxan.tw. or azaleptin.tw. or clozapine/ or clopine.tw. or clopsine.tw. or clozapine.tw. or Clozaril.tw. or denzapine.tw. or dorval.tw. or dozapine.tw. or elcrit.tw. or fazaclo.tw. or lapenax.tw. or leponex.tw. or lozapin.tw. or lozapine.tw. or sizopin.tw. or versacloz.tw. or zapen.tw. or zaponex.tw. or iloperidone/ or fanapt.tw. or fanaptum.tw. or zomaril.tw. or Lurasidone/ or Lurasidone.tw. or lurasidone/ or Latuda.tw. or lurasidone hydrochloride.tw. or olanzapine/ or anzatric.tw. or lanopin.tw. or lanzac.tw. or meltolan.tw. or midax.tw. or olace.tw. or oladay.tw. or olan.tw. or olandus.tw. or olanex.tw. or olanex.tw. or instab.tw. or olansek.tw. or olanzapine mylan.tw. or olanzapine pamoate.tw. or olanzapine pamoate dihydrate.tw. or olanzapine pamoate monohydrate.tw. or olapin.tw. or olazax.tw. or olazax disperzi.tw. or oleanz.tw. or olexar.tw. or oltal.tw. or olzap.tw. or onza.tw. or	210

© 2019 Yunusa I et al. *JAMA Network Open.*

Database	Search Strategy	Number of hits (As of May 2018)
	ozapin md.tw. or psychozap.tw. or relprevv.tw. or zalasta.tw. or zelta.tw. or zydis.tw. or zypadhera.tw. or zyprex*.tw. or paliperidone.tw. or Invega.tw. or invega sustenna.tw. or invega trinza.tw. or paliperidone palmitate .tw. or trevicta.tw. or xeplion.tw. or quetiapine/ or quetiapine fumarate.tw. or Seroquel.tw. or socalm.tw. or tienapine.tw. or risperidone/ or risperidone.tw. or belivon.tw. or consta.tw. or neripros.tw. or noprenia.tw. or riperidon.tw. or risolept.tw. or rispen.tw. or Risperdal.tw. or rispid.tw. or rispolet.tw. or rizodal.tw. or sequinan.tw. or zargus.tw. or zofredal.tw. or ziprasidone/ or ziprasidone.tw. or Geodon.tw. or zeldox.tw. or zeldrox.tw. or ziprasidone hydrochloride.tw. or ziprasidone hydrochloride monohydrate.tw. or ziprasidone mesylate.tw. or ziprasidone mesylate.tw. or zipsydon.tw.)<br>AND<br>(dementia/ OR dementia.tw. or alzheimers.tw.)<br>AND<br>(randomized controlled trial/ or randomized clinical trial.tw.)	
Cochrane Library	MeSH descriptor: [Dementia] explode all trees OR (Dementias or Senile Paranoid Dementia* or Familial Dementia*):ti,ab,kw (Word variations have been searched)<br>AND<br>MeSH descriptor: [Quetiapine Fumarate] OR MeSH descriptor: [Risperidone] OR MeSH descriptor: [Aripiprazole] OR MeSH descriptor: [Clozapine] OR MeSH descriptor: [Clozapine] explode all trees OR explode all trees OR (Risperdal Consta or Consta, Risperdal or Risperdal or ziprasidone or ziprasidone hydrochloride or ziprasidone hydrochloride, monohydrate or Aripiprazole OR Aripiprazol OR Abilify or Asenapine OR Asenapine maleate OR saphris OR Clozapine OR Clozaril OR Leponex OR Iloperidone OR Fanapt OR Lurasidone Hydrochloride OR Lurasidone OR Lurasidone HCL OR Latuda OR olanzapine OR Symbyax OR Zyprexa OR Paliperidone Palmitate OR Paliperidone Palmitate OR Paliperidone OR Invega or aripiprazole lauroxil) :ti,ab,kw (Word variations have been searched)	7
PsychINFO	(aripiprazole/ or aripiprazole monohydrate.tw. or abilify.tw. or asenapine/ or asenapine.tw. or asenapine maleate.tw. or saphris.tw. or sycrest.tw. or alemoxan.tw. or azaleptin.tw. or clozapine/ or clopine.tw. or clopsine.tw. or clozapine.tw. or Clozaril.tw. or denzapine.tw. or dorval.tw. or dozapine.tw. or elcrit.tw. or fazaclo.tw. or lapenax.tw. or leponex.tw. or lozapin.tw. or lozapine.tw. or sizopin.tw. or versacloz.tw. or zapen.tw. or zaponex.tw. or iloperidone/ or fanapt.tw. or	4

© 2019 Yunusa I et al. *JAMA Network Open.*

Database	Search Strategy	Number of hits (As of May 2018)
	fanaptum.tw. or zomaril.tw. or Lurasidone/ or Lurasidone.tw. or lurasidone/ or Latuda.tw. or lurasidone hydrochloride.tw. or olanzapine/ or anzatric.tw. or lanopin.tw. or lanzac.tw. or meltolan.tw. or midax.tw. or olace.tw. or oladay.tw. or olan.tw. or olandus.tw. or olanex.tw. or olanex.tw. or instab.tw. or olansek.tw. or olanzapine mylan.tw. or olanzapine pamoate.tw. or olanzapine pamoate dihydrate.tw. or olanzapine pamoate monohydrate.tw. or olapin.tw. or olazax.tw. or olazax disperzi.tw. or oleanz.tw. or olexar.tw. or oltal.tw. or olzap.tw. or onza.tw. or ozapin md.tw. or psychozap.tw. or relprevv.tw. or zalasta.tw. or zelta.tw. or zydis.tw. or zypadhera.tw. or zyprex*.tw. or paliperidone.tw. or Invega.tw. or invega sustenna.tw. or invega trinza.tw. or paliperidone palmitate .tw. or trevicta.tw. or xeplion.tw. or quetiapine/ or quetiapine fumarate.tw. or Seroquel.tw. or socalm.tw. or tienapine.tw. or risperidone/ or risperidone.tw. or belivon.tw. or consta.tw. or neripros.tw. or noprenia.tw. or riperidon.tw. or risolept.tw. or rispen.tw. or Risperdal.tw. or rispid.tw. or rispolet.tw. or rizodal.tw. or sequinan.tw. or zargus.tw. or zofredal.tw. or ziprasidone/ or ziprasidone.tw. or Geodon.tw. or zeldox.tw. or zeldrox.tw. or ziprasidone hydrochloride.tw. or ziprasidone hydrochloride monohydrate.tw. or ziprasidone mesylate.tw. or ziprasidone mesylate.tw. or zipsydon.tw.)<br>AND<br>(dementia/ OR dementia.tw. or alzheimers.tw.)<br>AND<br>(randomized controlled trial/ or randomized clinical trial.tw.)	

© 2019 Yunusa I et al. *JAMA Network Open.*

eTable2: Study and Patient Characteristics

Study	Study design	Study comparison(s)	Sample Size (N)	Mean/median age (years)	% Female	Trial duration (weeks)	Trial Setting	Clinical characteristics	MSSE at Baseline
Ballard, 2005[1]	Randomized, controlled, double blind trial	Quetiapine vs Placebo	93	83.8	79.6%	26	NH	AD with Agitation	N/R
Brodaty, 2003[2]	Randomized, controlled, double blind trial	Risperidone vs Placebo	345	83.0	71.9%	12	NH	AD, vascular, or mixed dementia	5.46
De Deyn, 2005[3]	Randomized, controlled, double blind trial	Aripiprazole vs Placebo	208	81.5	72.0%	10	Outpatient	AD with psychosis	4.35
De Deyn,2004[4]	Randomized, controlled, double blind trial	Olanzapine vs Placebo	652	76.6	75.0%	10	NH	AD with delusions and hallucination	13.7
De Deyn, 1999[5]	Randomized, controlled, double blind trial	Risperidone vs Placebo	344	81	58.0%	12	NH	AD with aggression, agitation	8.6
Deberdt, 2005 [6]	Randomized, controlled, double blind trial	Olanzapine vs Risperidone vs Placebo	494	78.3	65.2%	10	Outpatient, NH	Hallucinations, delusions	14.4
Katz, 1999[7]	Randomized, controlled, double blind trial	Risperidone vs Placebo	625	82.7	67.8%	12	NH	AD, vascular, or mixed dementia	6.6
Kurlan, 2007[8]	Randomized, controlled, double blind trial	Quetiapine vs Placebo	40	73.8	37.5%	10	NH, outpatient	AD with parkinsonian features, DLB, PD with dementia	18.1
Placeau, 2008[9]	Randomized, controlled, double blind trial	Quetiapine vs Placebo	40	82.2	65.0%	6	NH	AD with agitation, delusion, anxiety, apathy, irritability	14.4

© 2019 Yunusa I et al. *JAMA Network Open.*

Study	Study design	Study comparison(s)	Sample Size (N)	Mean/median age (years)	% Female	Trial duration (weeks)	Trial Setting	Clinical characteristics	MSSE at Baseline
Rainer, 2007[10]	Randomized, controlled, double blind trial	Quetiapine vs Risperidone	72	77.8	58.0%	8	Outpatient	Alzheimer's, vascular or mixed with delusions, hallucinations, agitation/ aggression, disinhibition and aberrant motor behavior	18.3
Schneider, 2006[11]	Randomized, controlled, double blind trial	Olanzapine vs Quetiapine vs Risperidone vs Placebo	421	77.9	56.0%	36	Outpatient	AD with psychosis, aggression, or agitation	15
Street, 2000[12]	Randomized, controlled, double blind trial	Olanzapine vs Placebo	206	82.8	61.2%	6	NH	AD with agitation, delusions, or hallucinations	6.7
Streim, 2008[13]	Randomized, controlled, double blind trial	Aripiprazole vs Placebo	256	83.0	76.1%	10	NH	AD with psychosis, delusions, hallucinations	13.6
Tariot, 2006[14]	Randomized, controlled, double blind trial	Quetiapine vs Placebo	284	83.2	73.0%	10	NH	AD, vascular dementia or mixed with psychosis	12.8
Zhong, 2007[15]	Randomized, controlled, double blind trial	Quetiapine vs Placebo	333	83.0	74.0%	10	NH	AD, vascular or mixed dementia with agitation	5.3
Mintzer, 2006[16]	Randomized, controlled, double blind trial	Risperidone vs Placebo	473	83.3	77.0%	8	NH	AD, Vascular dementia with psychosis	13.2
Mintzer, 2007[17]	Randomized, controlled, double blind trial	Aripiprazole vs Placebo	487	82.5	79.0%	10	NH	AD with psychotic symptoms of delusions or hallucinations	N/R

Abbreviations: AD: Alzheimer's Disease; NH: Nursing Home; N/R: Not reported; MMSE: Mini-Mental State Examination

© 2019 Yunusa I et al. *JAMA Network Open.*

eTable 3: Risk of Bias Assessment

Author	Sequence generation (Selection bias)	Allocation Concealment (Selection bias)	Blinding of participants and personnel (Performance bias)	Blinding of outcome assessment (detection bias)	Incomplete outcome data (attrition bias)	Selective outcome reporting (reporting bias)	Overall risk of bias
Ballard, 2005	Low	Low	Low	Low	Low	Low	Low
Brodaty, 2003	Low	Low	Low	Low	Low	Low	Low
De Deyn, 2005	Unclear	Low	Low	Low	Low	Low	Medium
De Deyn,2004	Unclear	Low	Low	Low	Low	Low	Medium
De Deyn, 1999	Unclear	Low	Low	Low	Low	Low	Medium
Deberdt, 2005	Unclear	Low	Low	Low	Low	Low	Medium
Katz, 1999	Low	Low	Low	Low	Low	Low	Low
Kurlan, 2007	Low	Low	Low	Low	Low	Low	Low
Placeau, 2008	Unclear	Low	Low	Low	Low	Low	Medium
Rainer, 2007	Low	Low	Unclear	Low	Low	Low	Medium
Schneider, 2006	Low	Low	Low	Low	Low	Low	Low
Street, 2000	Low	Low	Low	Low	Low	Low	Low
Streim, 2008	Unclear	Low	Low	Low	Low	Low	Medium
Tariot, 2006	Low	Low	Low	Low	Low	Low	Low
Zhong, 2007	Low	Low	Low	Low	Low	Low	Low
Mintzer, 2006	Low	Low	Low	Low	Low	Low	Low
Mintzer, 2007	Unclear	Low	Low	Low	Low	Low	Medium

© 2019 Yunusa, I, et al. *JAMA Network Open.*

eTable 4: Inconsistency tests based on loop-specific approach

eTable 4A: Inconsistency plot for Neuropsychiatric Inventory (NPI)

Loop	IF	seIF	z_value	p_value	CI_95	Loop_Heterog_tau2
1-3-4	0.227	0.267	0.848	0.396	(0.00,0.75)	0.000
3-4-5	0.146	0.315	0.463	0.643	(0.00,0.76)	0.000
1-4-5	0.074	0.235	0.315	0.753	(0.00,0.54)	0.001
1-3-5	0.065	0.246	0.265	0.791	(0.00,0.55)	0.015

For the continuous outcomes, inconsistency factors (IF) are the differences of standardized mean differences (SMDs). seIF = standard error of the IF. CI_95 = 95% confidence interval. Loop_Heterog_tau2 = loop-specific heterogeneity. All loops were consistent ($p > 0.1$) indicating lack of evidence of inconsistency in the network. In column named loop; 1 = Placebo, 2 = Aripiprazole, 3 = Olanzapine, 4 = Quetiapine, and 5 = Risperidone.

© 2019 Yunusa, I, et al. *JAMA Network Open.*

eTable 4B: Inconsistency plot for Death

Evaluation of inconsistency using loop-specific heterogeneity estimates:

Loop	ROR	z_value	p_value	CI_95	Loop_Heterog_tau2
1 3 4	5.426	1.218	0.223	(1.00,82.48)	0.000
3 4 5	5.298	0.690	0.490	(1.00,603.19)	0.000
1 4 5	2.054	0.635	0.525	(1.00,18.93)	0.000
1 3 5	1.073	0.083	0.934	(1.00,5.60)	0.000

For the dichotomous outcomes, inconsistency factors (IF) are the ratios of odds ratios (RORs). CI_95 = 95% confidence interval. Loop_Heterog_tau2 = loop-specific heterogeneity. All loops were consistent ($p > 0.1$) indicating lack of evidence of inconsistency in the network. In column named loop; 1 = Placebo, 2 = Aripiprazole, 3 = Olanzapine, 4 = Quetiapine, and 5 = Risperidone.

© 2019 Yunusa, I, et al. *JAMA Network Open.*

eTable 4C: Inconsistency plot for cerebrovascular adverse events (CVAE)

Evaluation of inconsistency using loop-specific heterogeneity estimates:

Loop	ROR	z_value	p_value	CI_95	Loop_Heterog_tau2
1 4 5	4.405	1.029	0.303	(1.00,74.15)	0.000
1 3 4	1.992	0.405	0.686	(1.00,56.22)	0.000
3 4 5	1.796	0.239	0.811	(1.00,217.57)	0.000
1 3 5	1.781	0.466	0.641	(1.00,20.18)	0.000

For the dichotomous outcomes, inconsistency factors (IF) are the ratios of odds ratios (RORs). CI_95 = 95% confidence interval. Loop_Heterog_tau2 = loop-specific heterogeneity. All loops were consistent ($p > 0.1$) indicating lack of evidence of inconsistency in the network. In column named loop; 1 = Placebo, 2 = Aripiprazole, 3 = Olanzapine, 4 = Quetiapine, and 5 = Risperidone.

© 2019 Yunusa, I, et al. *JAMA Network Open.*

eTable 5: Inconsistency test based on side-splitting approach

eTable 5A: Inconsistency plot for Neuropsychiatric Inventory (NPI)

Side	*Direct*		*Indirect*		*Difference*		
	Coef.	Std. Err.	Coef.	Std. Err.	Coef.	Std. Err.	P>z
1 2	.	.	.	.	.	.	.
1 3	.	.	.	.	.	.	.
1 4	-.06696	.0926224	-.1929519	.2758251	.1259919	.2876401	0.661
1 5	-.0215682	.103871	.1044251	.2717902	-.1259933	.2876411	0.661
3 4	-.1394471	.2079974	.0653145	.1130288	-.2047617	.2260545	0.365
3 5	.0833758	.0994069	.2093692	.2793801	-.1259933	.2876411	0.661
4 5	-.0284746	.1652412	.1435115	.1463277	-.1719862	.1996639	0.389

In column named side; 1 = Placebo, 2 = Aripiprazole, 3 = Olanzapine, 4 = Quetiapine, and 5 = Risperidone. For each contrast, the direct and indirect estimates (i.e. standardized mean differences and represented as Coef in the table) and the respective inconsistency factor (difference between direct and indirect estimates) are provided along with their standard errors (SE). P-values smaller than 0.10 correspond to statistically significant inconsistency factors.

Coef = coefficient; Std Err = standard error.

© 2019 Yunusa, I, et al. *JAMA Network Open.*

eTable 5B: Inconsistency plot for Death

Side	*Direct*		*Indirect*		*Difference*		
	Coef.	Std. Err.	Coef.	Std. Err.	Coef.	Std. Err.	P>z
1 2	.	.	.	.	.	.	.
1 3	.	.	.	.	.	.	.
1 4	.5097396	.412655	.1645624	2.03216	.3451771	2.070432	0.868
1 5	.274329	.2764425	.6194299	2.055537	-.3451009	2.07084	0.868
3 4	.2898394	.8136067	-.2396464	.6458957	.5294858	.8885014	0.551
3 5	-.4326372	.5697664	-.1099773	.5760269	-.3226599	.7092432	0.649
4 5	-.6319199	.9677029	-.1338403	.4991546	-.4980796	1.014631	0.623

In column named side; 1 = Placebo, 2 = Aripiprazole, 3 = Olanzapine, 4 = Quetiapine, and 5 = Risperidone. For each contrast, the direct and indirect estimates (i.e. log-odds ratios and represented as Coef in the table) and the respective inconsistency factor (difference between direct and indirect estimates) are provided along with their standard errors (SE). P-values smaller than 0.10 correspond to statistically significant inconsistency factors.

Coef = coefficient; Std Err = standard error.

© 2019 Yunusa, I, et al. *JAMA Network Open.*

eTable 5C: Inconsistency plot for cerebrovascular adverse events (CVAE)

Side	*Direct*		*Indirect*		*Difference*		
	Coef.	Std. Err.	Coef.	Std. Err.	Coef.	Std. Err.	P>z
1 2	.	.	.	.	.	.	.
1 3	.	.	.	.	.	.	.
1 4	.2132722	.6020222	1.509528	2.067684	-1.296256	2.135803	0.544
1 5	1.400474	.4713644	.1035713	2.101756	1.296903	2.136221	0.544
3 4	-.6681624	1.157802	-1.416172	.9048707	.7480092	1.353347	0.580
3 5	-.2346004	.5702288	.4240575	.9820908	-.6586579	1.024169	0.520
4 5	.3279686	1.051107	1.298427	.7317245	-.970458	1.09722	0.376

In column named side; 1 = Placebo, 2 = Aripiprazole, 3 = Olanzapine, 4 = Quetiapine, and 5 = Risperidone. For each contrast, the direct and indirect estimates (i.e. log-odds ratios and represented as Coef in the table) and the respective inconsistency factor (difference between direct and indirect estimates) are provided along with their standard errors (SE). P-values smaller than 0.10 correspond to statistically significant inconsistency factors.

Coef = coefficient; Std Err = standard error.

© 2019 Yunusa, I, et al. *JAMA Network Open.*

eFigure 1: PRISMA Study Selection Flowchart

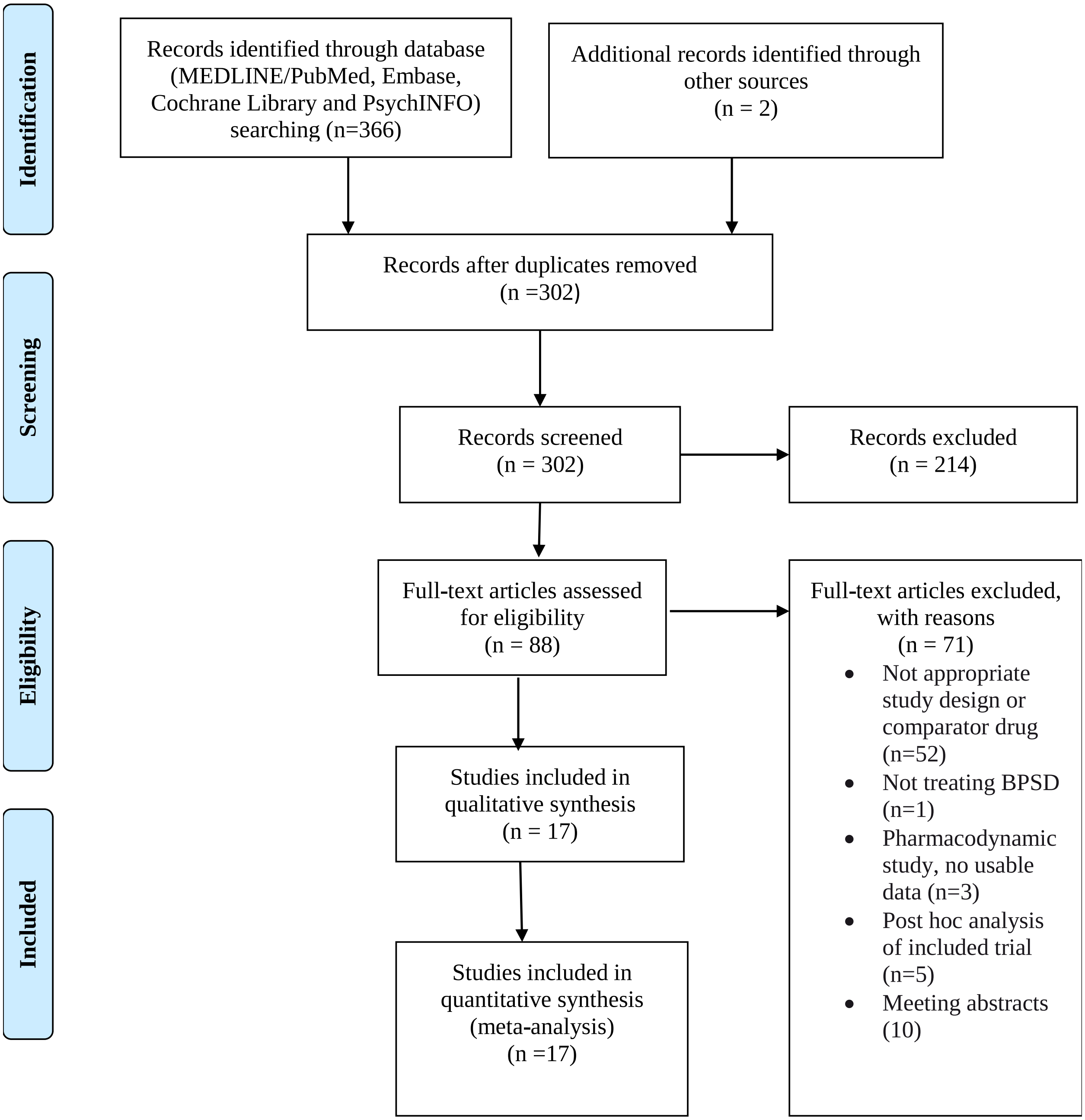

Abbreviation: BPSD: Behavioral and Psychological Symptoms of Dementia

© 2019 Yunusa, I, et al. *JAMA Network Open.*

eFigure 2: Network Plots for Secondary Outcomes

2A: Network diagram for Behavioral and Psychological Symptoms of Dementia (BPRS)

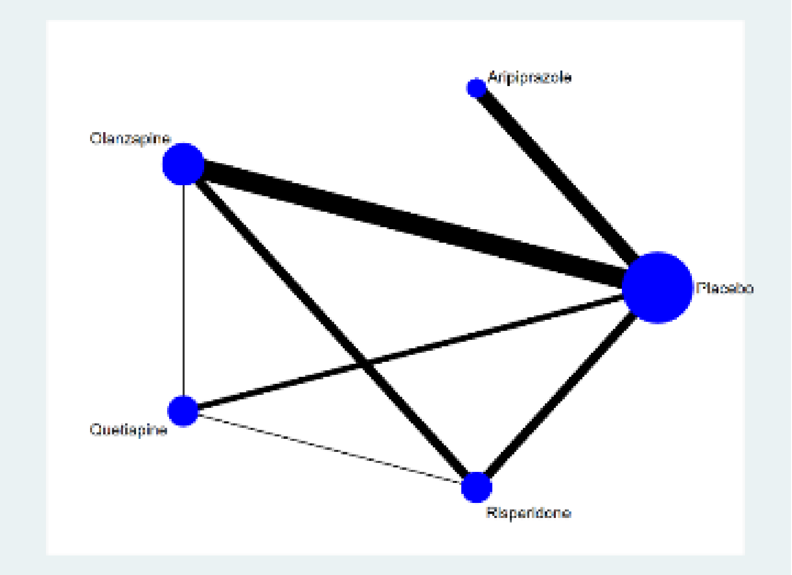

The width of the lines is proportional to the number of trials comparing every pair of treatment, and the size of every node is proportional to the number of randomized participants (sample size)

eFigure 2B: Network diagram for Cohen-Mansfield Agitation Inventory (CMAI)

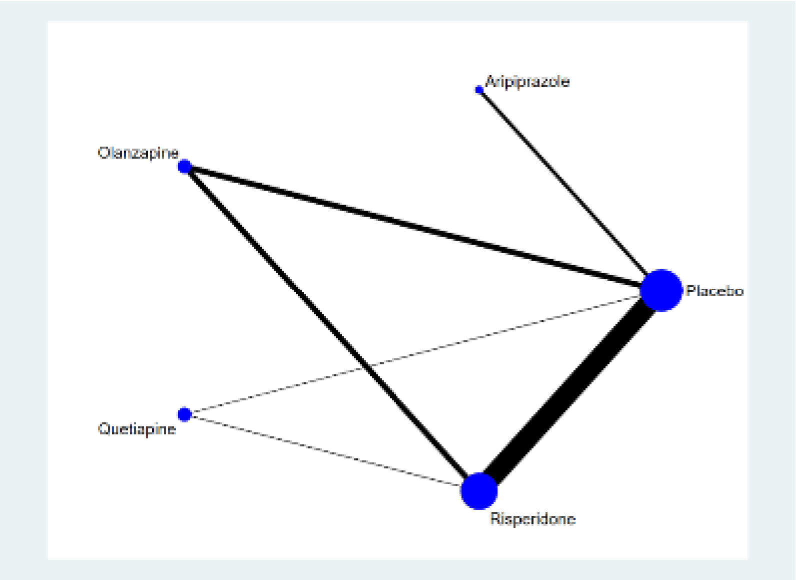

The width of the lines is proportional to the number of trials comparing every pair of treatment, and the size of every node is proportional to the number of randomized participants (sample size)

© 2019 Yunusa, I, et al. *JAMA Network Open.*

eFigure 2C: Network diagram for extrapyramidal signs/symptoms (EPS)

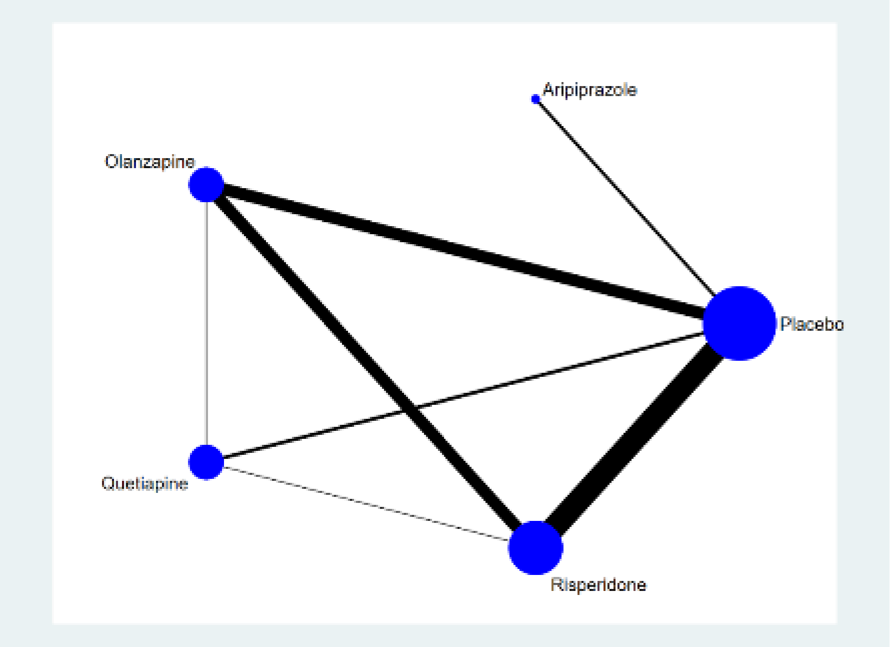

The width of the lines is proportional to the number of trials comparing every pair of treatment, and the size of every node is proportional to the number of randomized participants (sample size)

© 2019 Yunusa, I, et al. *JAMA Network Open.*

eFigure 2D: Network diagram for Somnolence or sedation

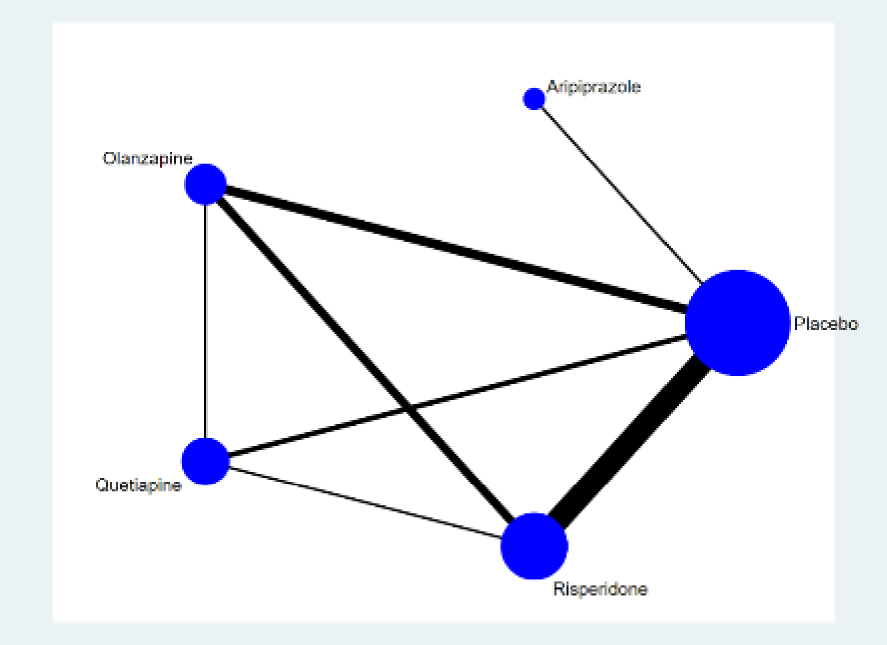

The width of the lines is proportional to the number of trials comparing every pair of treatment, and the size of every node is proportional to the number of randomized participants (sample size)

© 2019 Yunusa, I, et al. *JAMA Network Open.*

eFigure 2E: Network diagram for Fall, fracture or injury

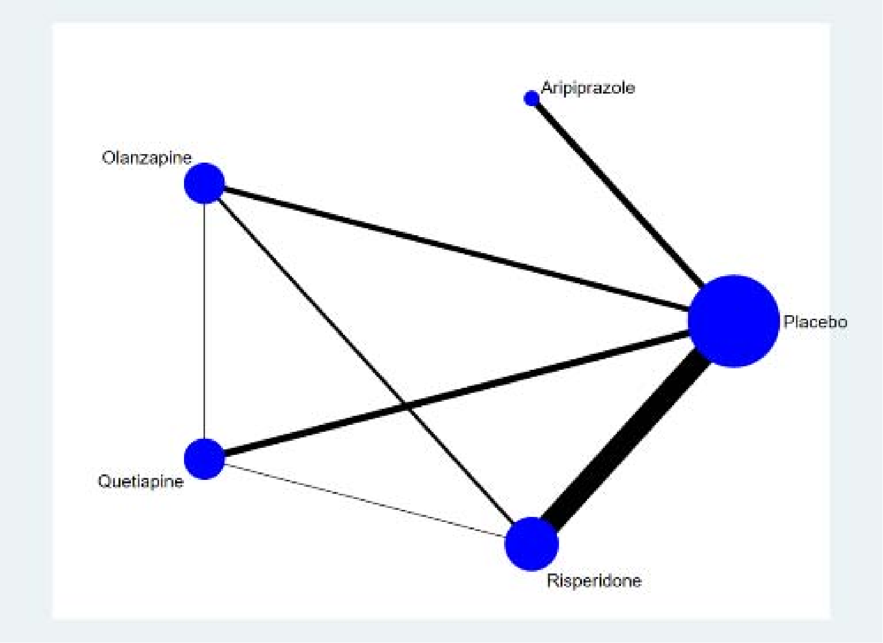

The width of the lines is proportional to the number of trials comparing every pair of treatment, and the size of every node is proportional to the number of randomized participants (sample size)

© 2019 Yunusa, I, et al. *JAMA Network Open.*

eFigure 2F: Network diagram for urinary tract infection (UTI) or incontinence

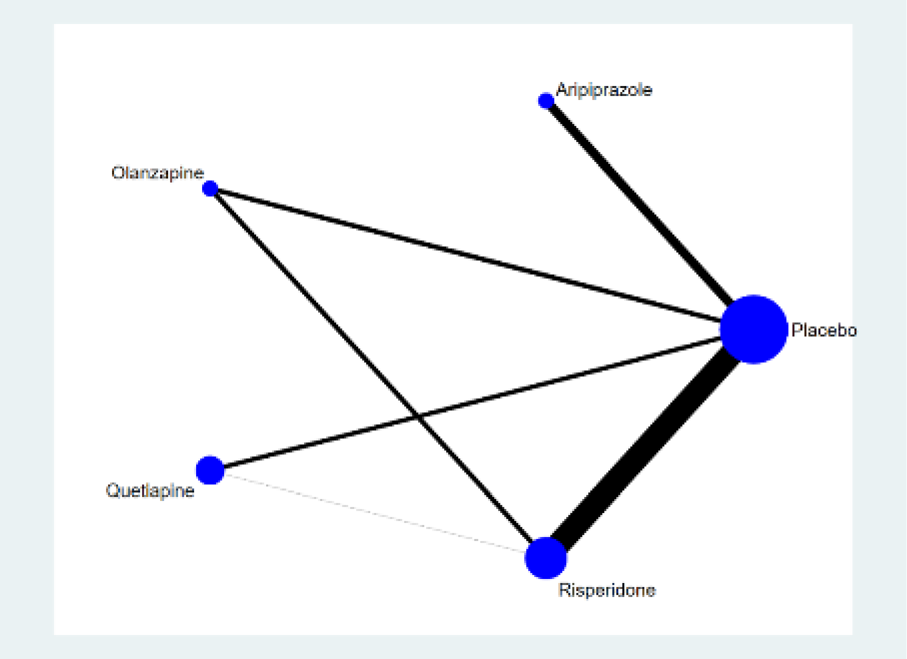

The width of the lines is proportional to the number of trials comparing every pair of treatment, and the size of every node is proportional to the number of randomized participants (sample size)

eFigure 3: Surface under the cumulative ranking curves (SUCRAs) showing the percentage of effectiveness against an imaginary treatment that should rank as the most effective without uncertainty. The value of SUCRA would be 100% for the most effective treatment with no uncertainty and 0% for the worst.

3A SUCRA for Neuropsychiatric Inventory (NPI)

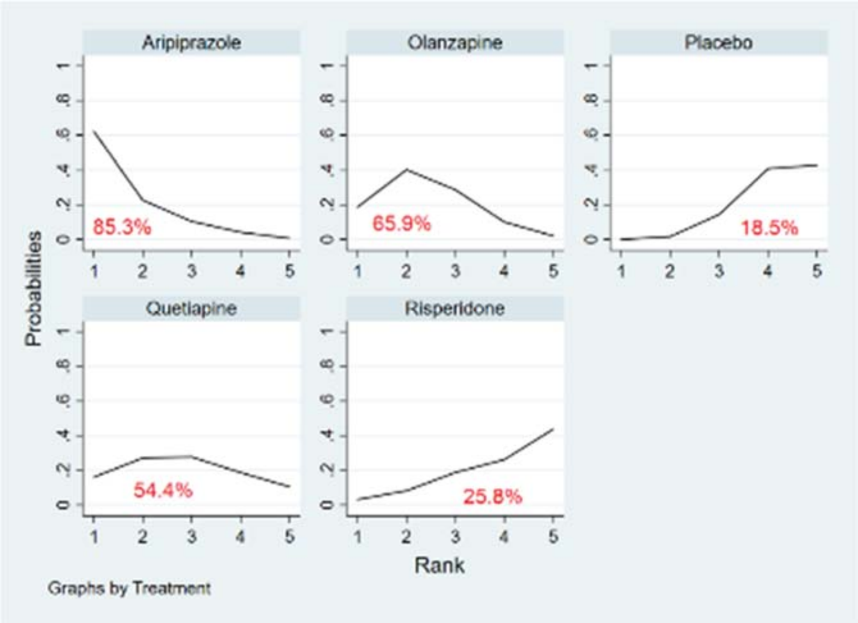

© 2019 Yunusa, I, et al. *JAMA Network Open.*

eFigure 3B SUCRA for Behavioral and Psychological Symptoms of Dementia (BPRS)

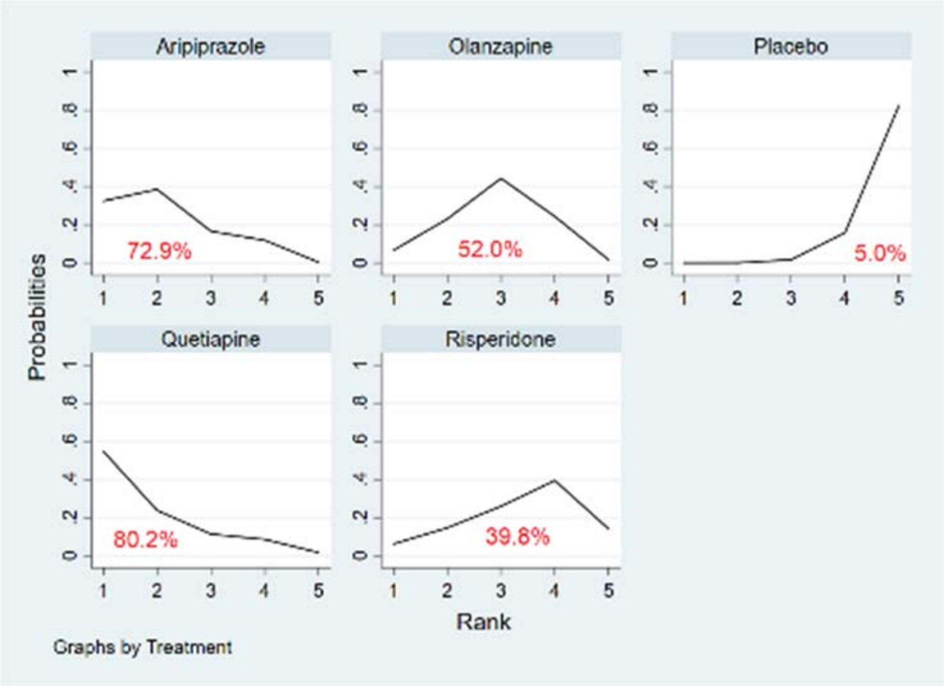

© 2019 Yunusa, I, et al. *JAMA Network Open.*

eFigure 3C SUCRA for Cohen-Mansfield Agitation Inventory (CMAI)

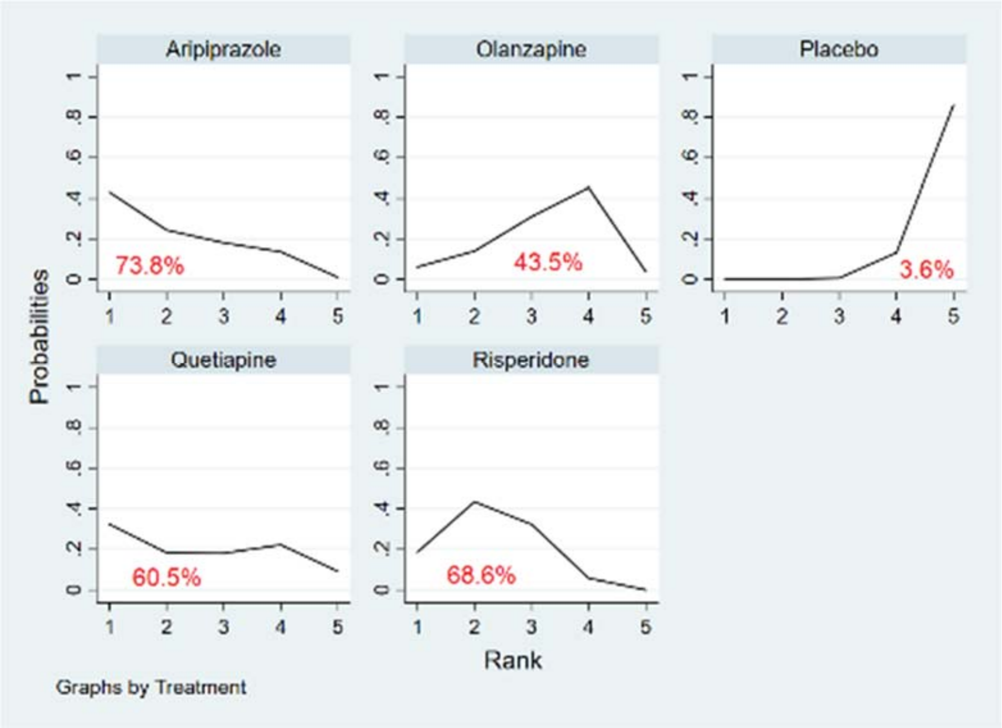

© 2019 Yunusa, I, et al. *JAMA Network Open.*

eFigure 3D SUCRA for Death

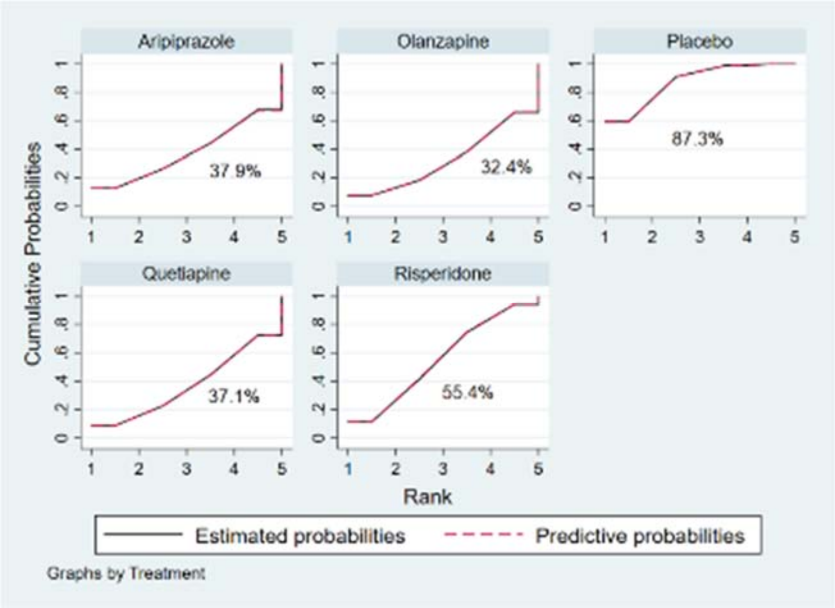

© 2019 Yunusa, I, et al. *JAMA Network Open.*

eFigure 3E SUCRA for Cerebrovascular adverse events (CVAE)

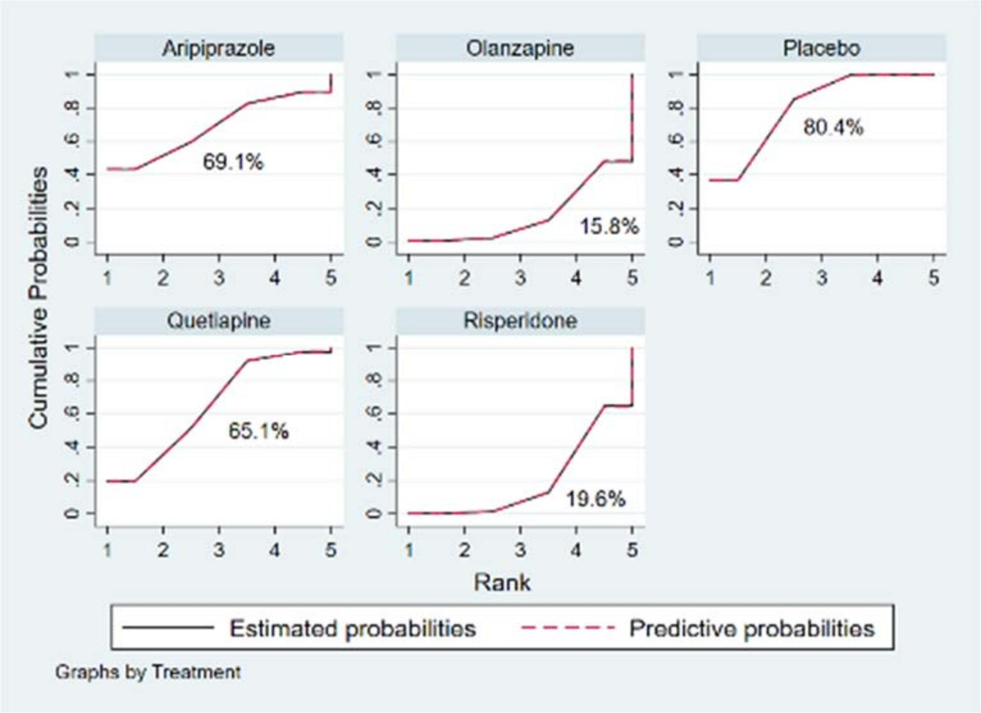

© 2019 Yunusa, I, et al. *JAMA Network Open.*

eFigure 3F SUCRA for Extrapyramidal signs/symptoms (EPS)

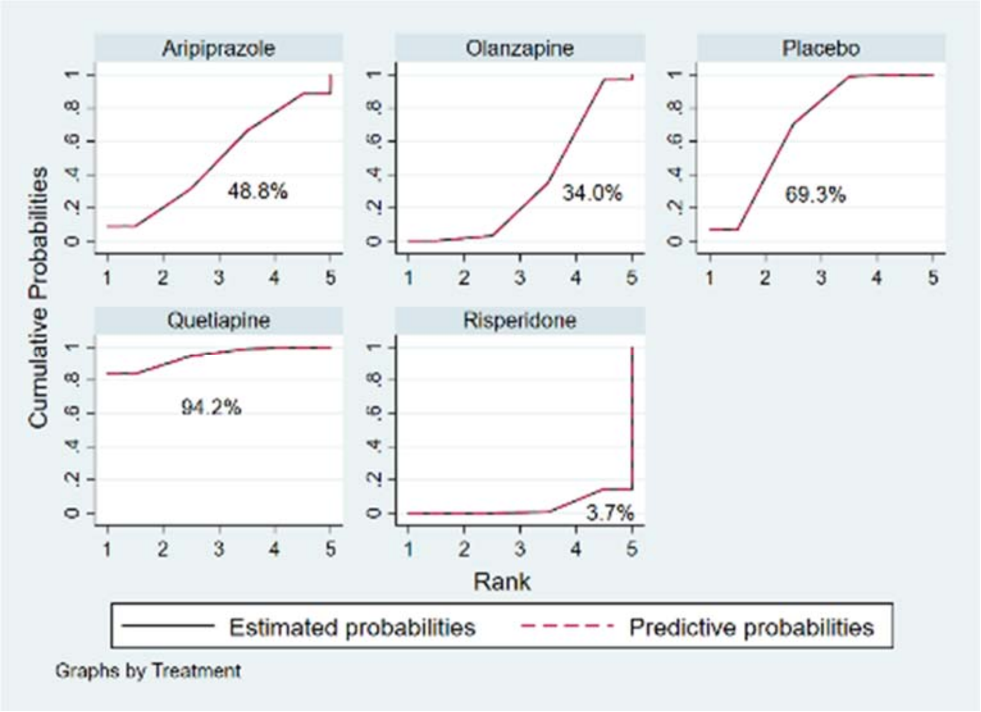

© 2019 Yunusa, I, et al. *JAMA Network Open.*

eFigure 3G SUCRA for Somnolence or sedation

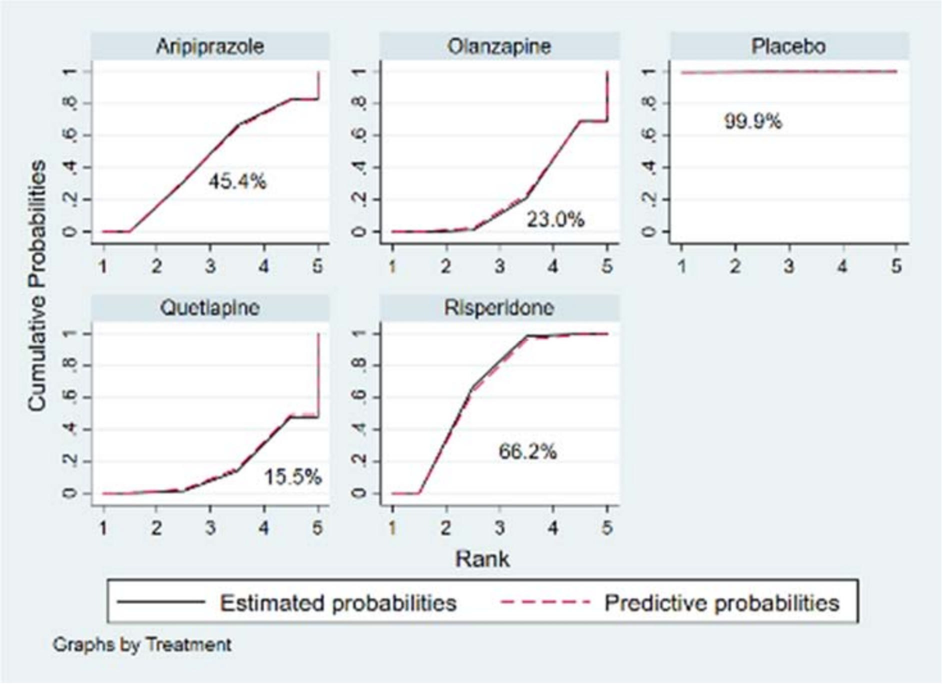

© 2019 Yunusa, I, et al. *JAMA Network Open.*

eFigure 3H SUCRA for Fall, fracture or injury

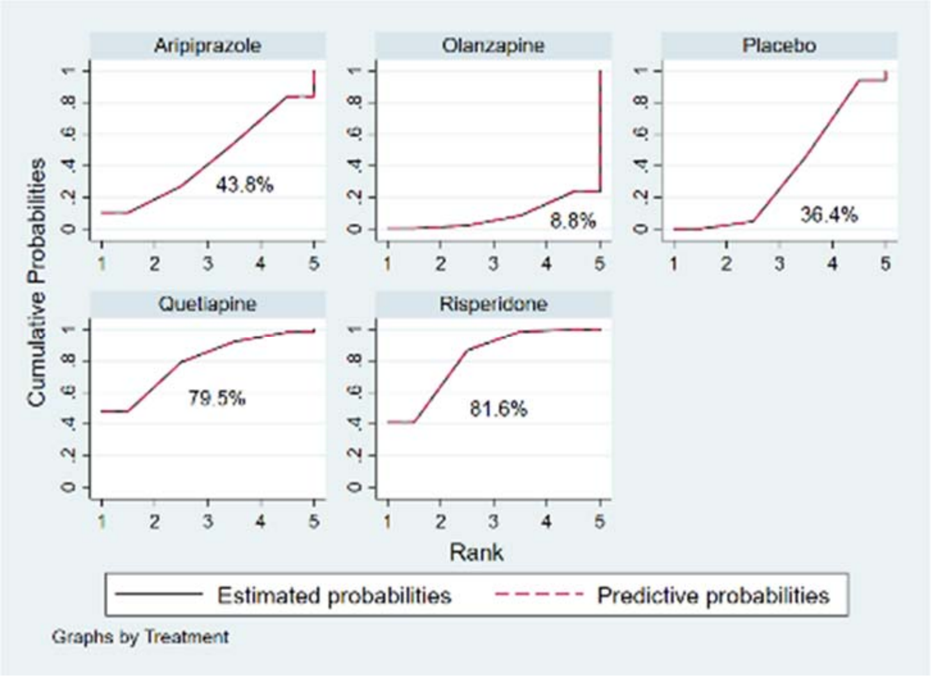

© 2019 Yunusa, I, et al. *JAMA Network Open.*

eFigure 3I SUCRA for Urinary incontinence or urinary tract infection (UTI)

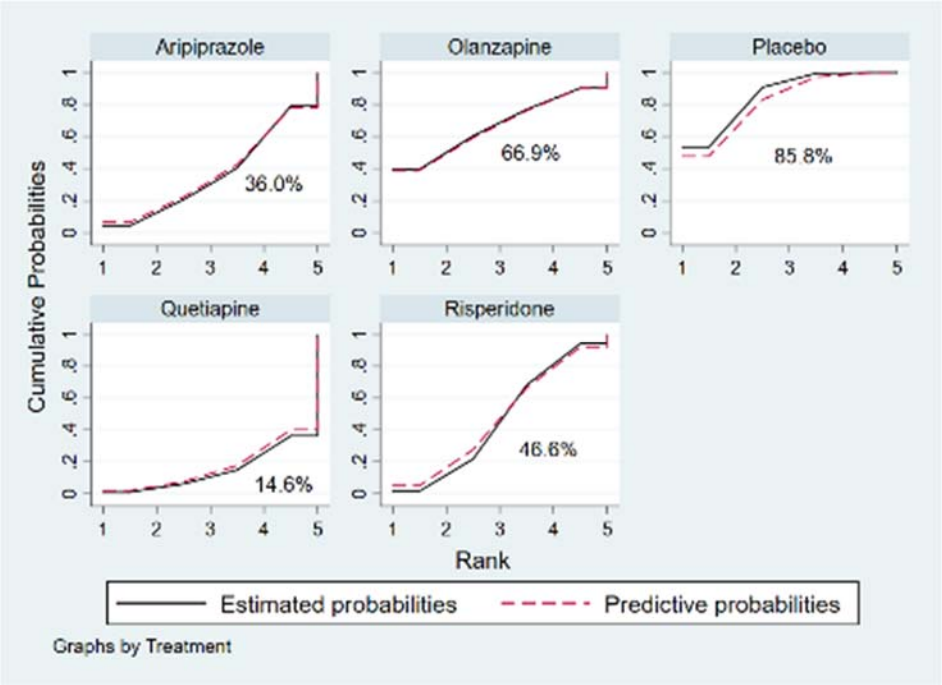

© 2019 Yunusa, I, et al. *JAMA Network Open.*

eFigure 4: Interval Plots

4A Interval plot for Behavioral and Psychological Symptoms of Dementia (BPRS)

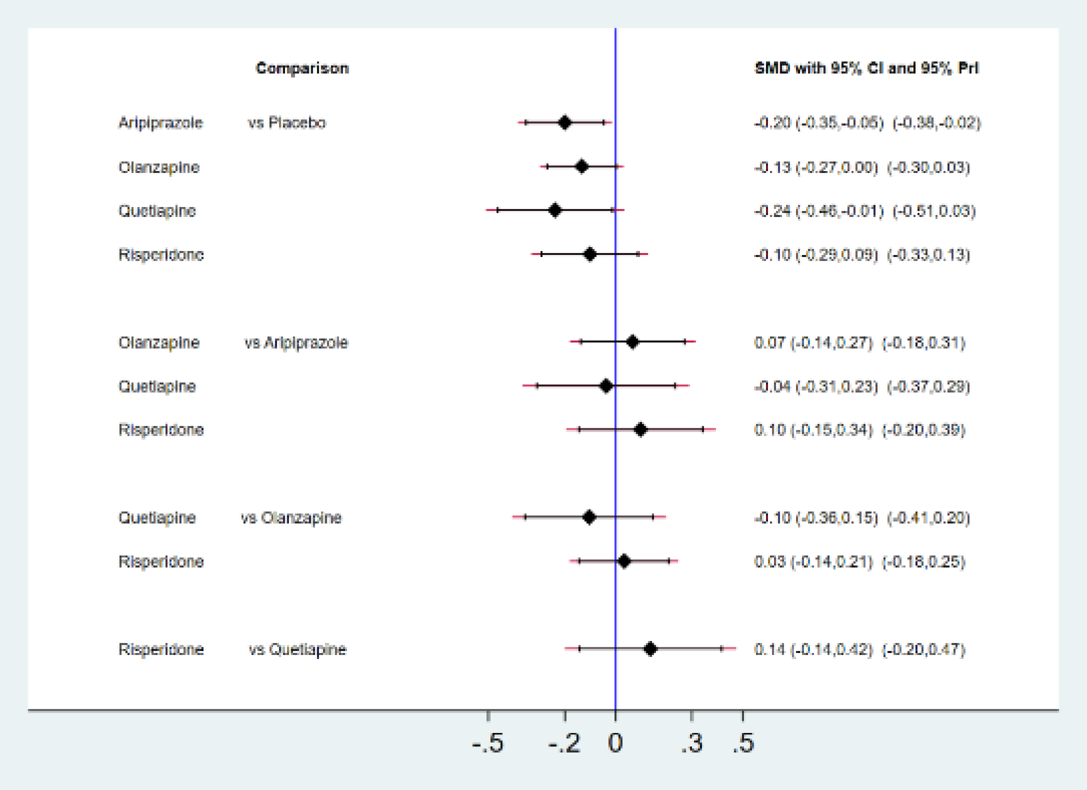

The black solid lines represent the confidence intervals for the standardized mean difference for each comparison and the red dashed lines the respective predictive intervals. The blue line is the line of no effect (difference equal to 0).

eFigure 4B Interval plot for Cohen-Mansfield Agitation Inventory (CMAI)

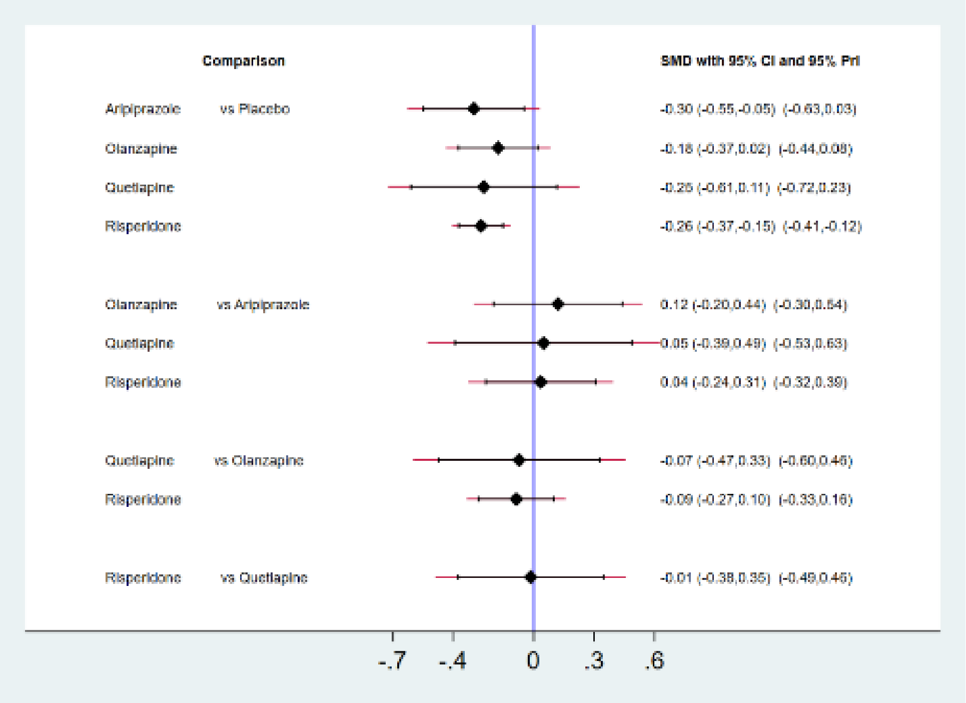

The black solid lines represent the confidence intervals for the standardized mean difference for each comparison and the red dashed lines the respective predictive intervals. The blue line is the line of no effect (difference equal to 0).

eFigure 4C Interval plot for Death

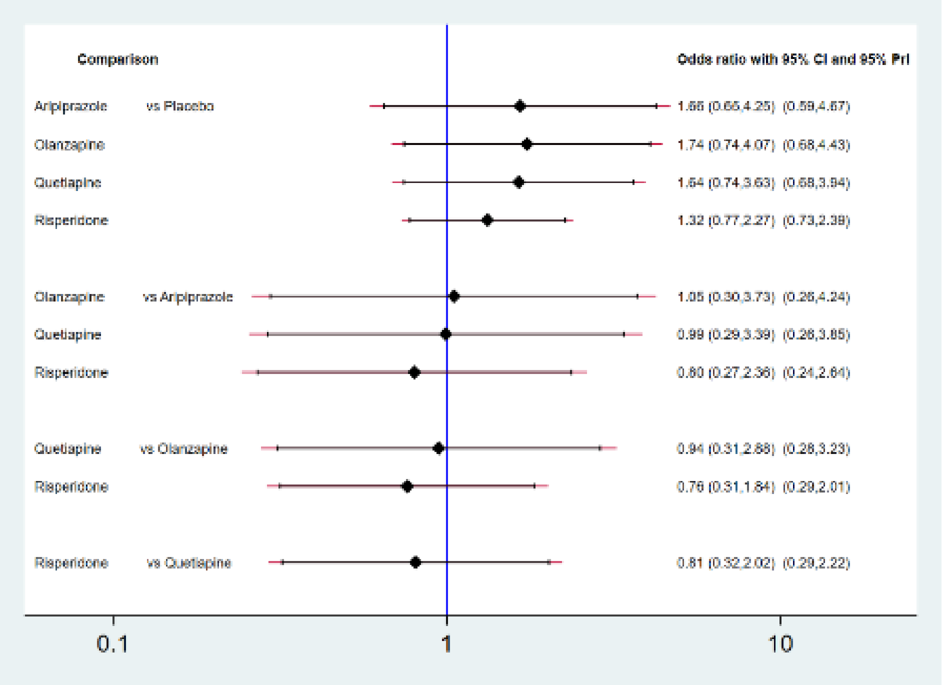

The black solid lines represent the confidence intervals for the Odds Ratios for each comparison and the red dashed lines the respective predictive intervals. The blue line is the line of no effect (ratio equal to 1).

© 2019 Yunusa, I, et al. *JAMA Network Open.*

eFigure 4D Interval Plot for cerebrovascular adverse events (CVAE)

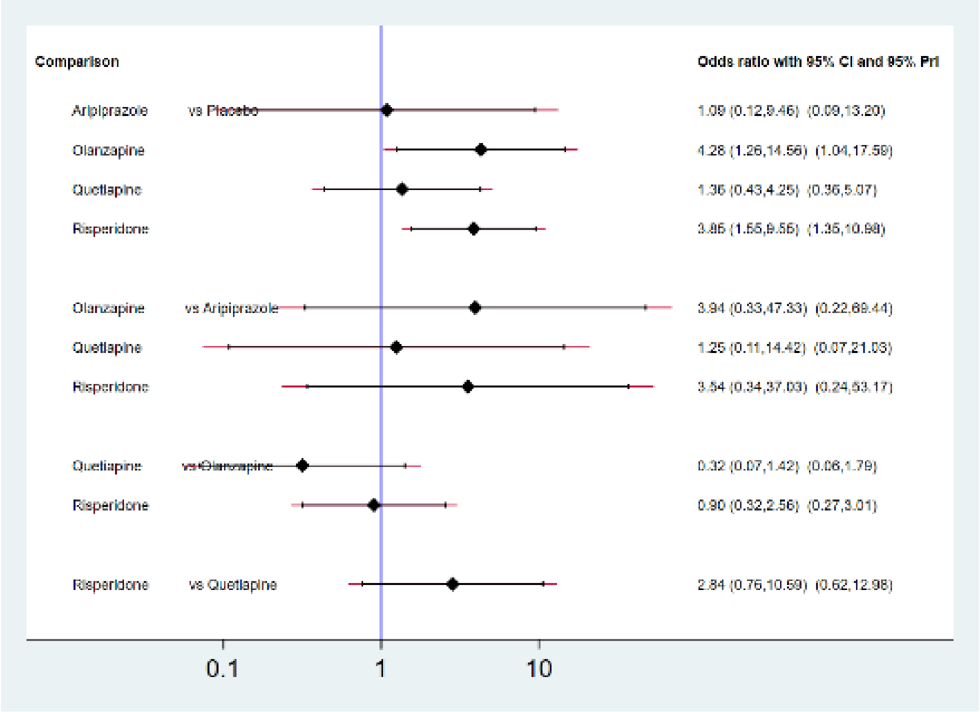

The black solid lines represent the confidence intervals for the Odds Ratios for each comparison and the red dashed lines the respective predictive intervals. The blue line is the line of no effect (ratio equal to 1).

© 2019 Yunusa, I, et al. *JAMA Network Open.*

eFigure 4E Interval Plot for Extrapyramidal signs/symptoms (EPS)

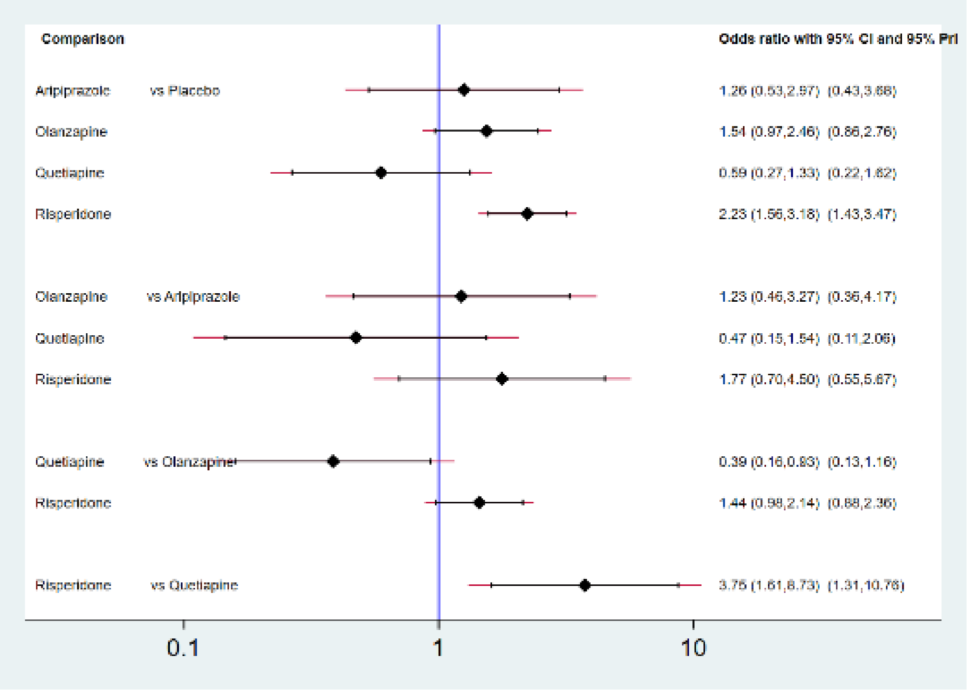

The black solid lines represent the confidence intervals for the Odds Ratios for each comparison and the red dashed lines the respective predictive intervals. The blue line is the line of no effect (ratio equal to 1).

© 2019 Yunusa, I, et al. *JAMA Network Open.*

eFigure 4F Interval Plot for Somnolence or sedation

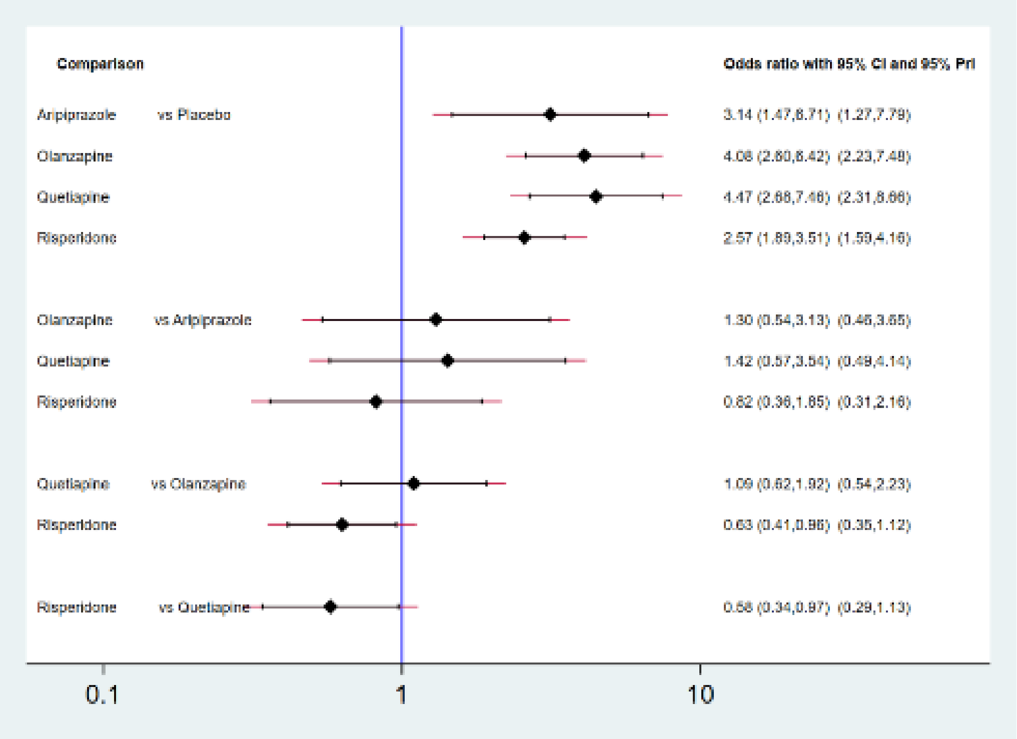

The black solid lines represent the confidence intervals for the Odds Ratios for each comparison and the red dashed lines the respective predictive intervals. The blue line is the line of no effect (ratio equal to 1).

© 2019 Yunusa, I, et al. *JAMA Network Open.*

eFigure 4G Interval Plot for Fall, fracture or injury

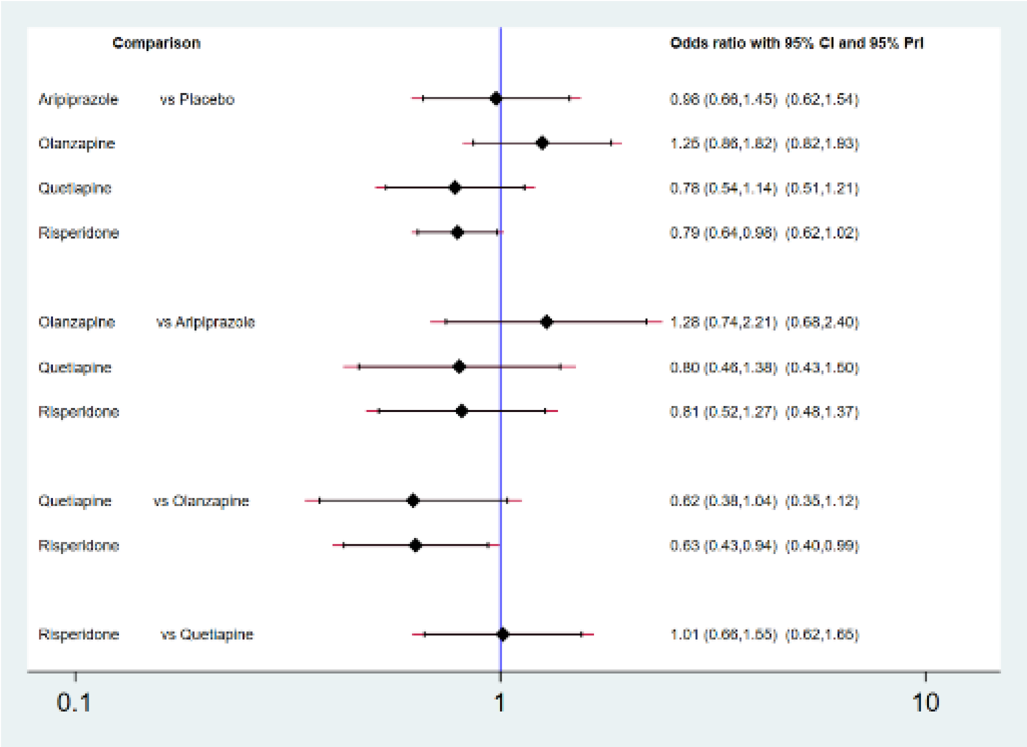

The black solid lines represent the confidence intervals for the Odds Ratios for each comparison and the red dashed lines the respective predictive intervals. The blue line is the line of no effect (ratio equal to 1).

© 2019 Yunusa, I, et al. *JAMA Network Open.*

eFigure 4H. Interval Plot for urinary tract infection (UTI) or urinary incontinence

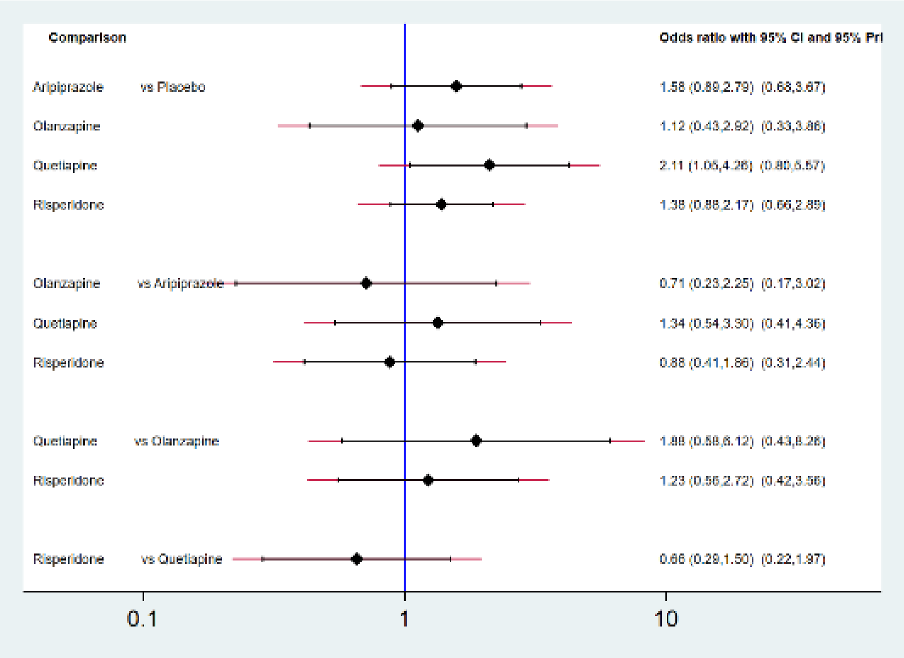

The black solid lines represent the confidence intervals for the Odds Ratios for each comparison and the red dashed lines the respective predictive intervals. The blue line is the line of no effect (ratio equal to 1).

© 2019 Yunusa, I, et al. *JAMA Network Open.*

eFigure 5: Comparison-adjusted funnel plots for primary outcomes involving all studies comparing all AAPs vs. Placebo.

eFigure 5A: Comparison adjusted funnel plot for Neuropsychiatric Inventory (NPI)[a]

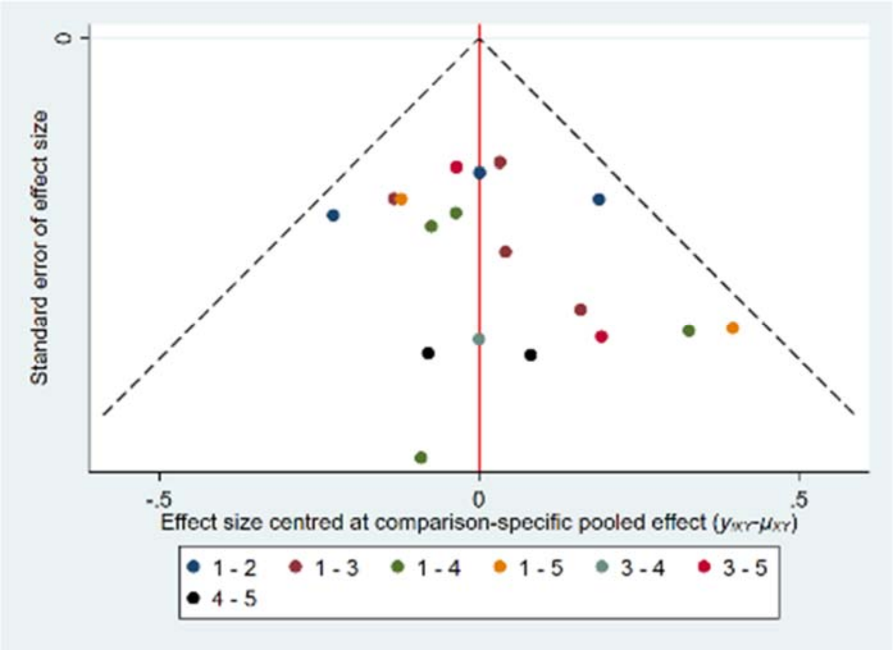

[a]Where; 1 = Placebo, 2 = Aripiprazole, 3 = Olanzapine, 4 = Quetiapine, and 5 = Risperidone.

© 2019 Yunusa, I, et al. *JAMA Network Open.*

eFigure 5B: Comparison adjusted funnel plot for Death[b]

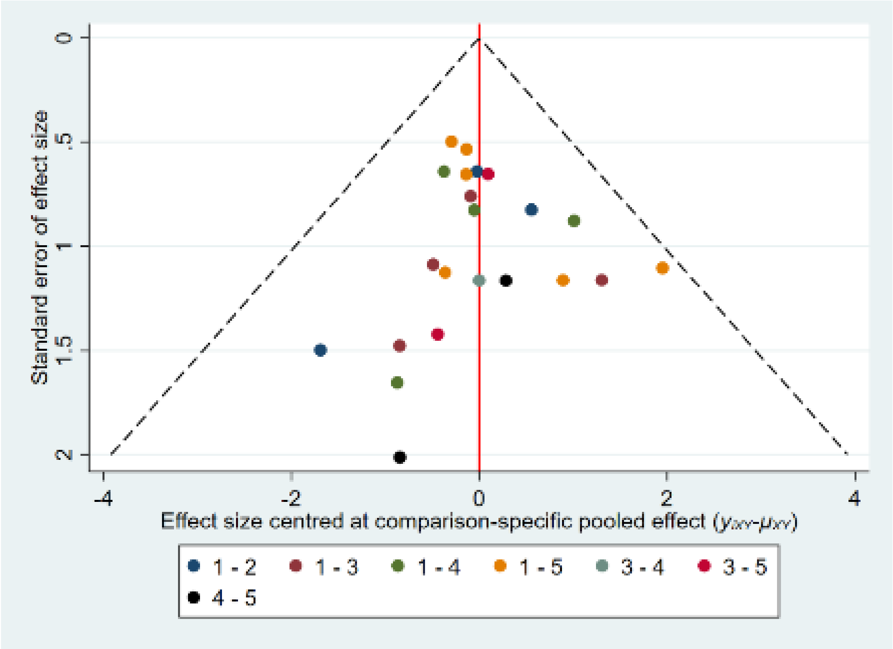

[b]Where; 1 = Placebo, 2 = Aripiprazole, 3 = Olanzapine, 4 = Quetiapine, and 5 = Risperidone.

© 2019 Yunusa, I, et al. *JAMA Network Open.*

eFigure 5C: Comparison adjusted funnel plot for cerebrovascular adverse events (CVAE)[c]

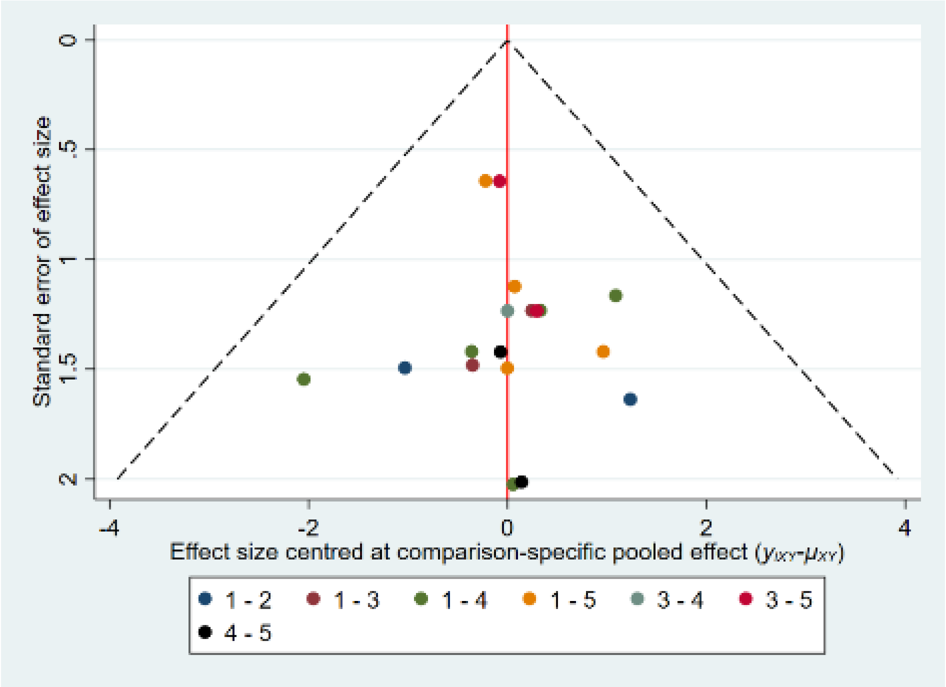

[c]Where; 1 = Placebo, 2 = Aripiprazole, 3 = Olanzapine, 4 = Quetiapine, and 5 = Risperidone.

© 2019 Yunusa, I, et al. *JAMA Network Open.*

eFigure 6: Sensitivity analysis removing studies with small sample size

eFigure 6A: Interval plot for Neuropsychiatric Inventory (NPI) when studies with a sample size of 100 or less were removed

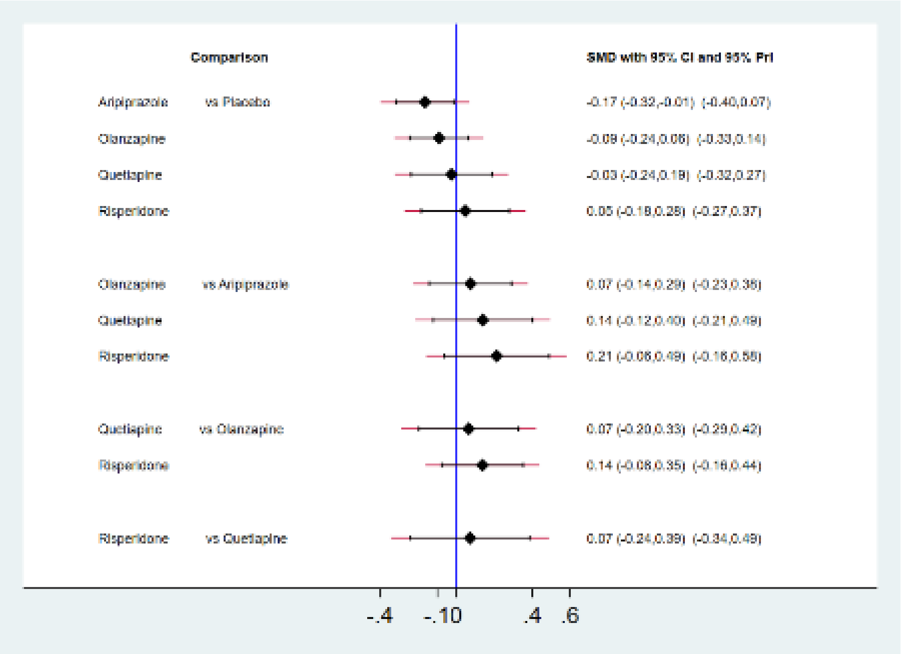

The black solid lines represent the confidence intervals for the Odds Ratios for each comparison and the red dashed lines the respective predictive intervals. The blue line is the line of no effect (ratio equal to 1).

© 2019 Yunusa, I, et al. *JAMA Network Open.*

eFigure 6B: Interval plot for Death when studies with a sample size of 100 or less were removed

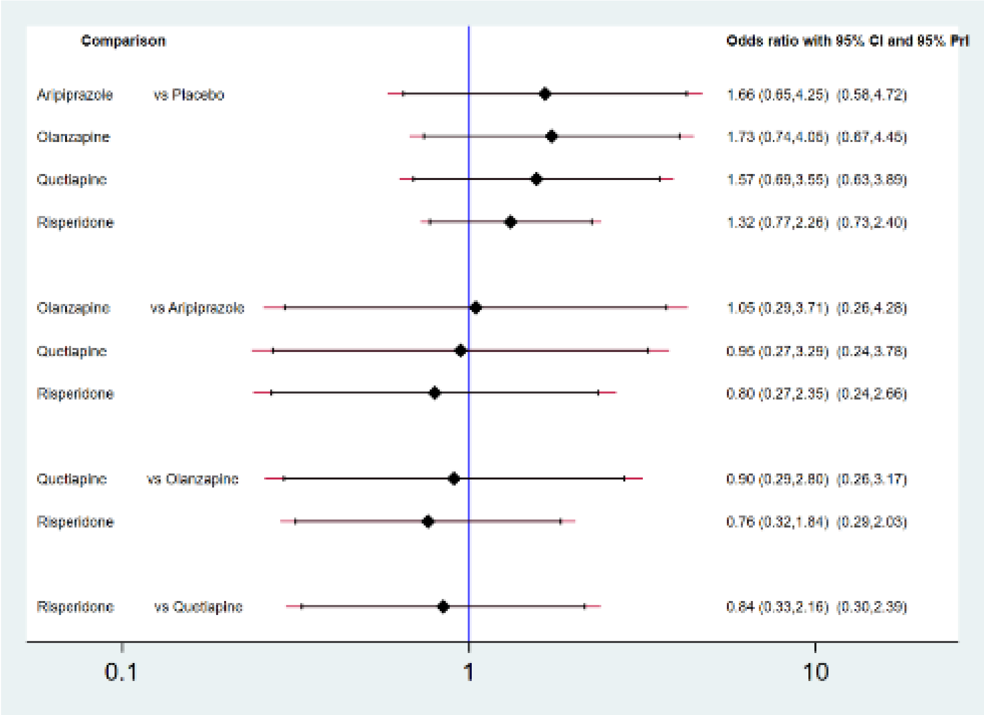

The black solid lines represent the confidence intervals for the Odds Ratios for each comparison and the red dashed lines the respective predictive intervals. The blue line is the line of no effect (ratio equal to 1).

© 2019 Yunusa, I, et al. *JAMA Network Open.*

eFigure 6C: Interval plot for cerebrovascular adverse events (CVAE) when studies with a sample size of 100 or less were removed

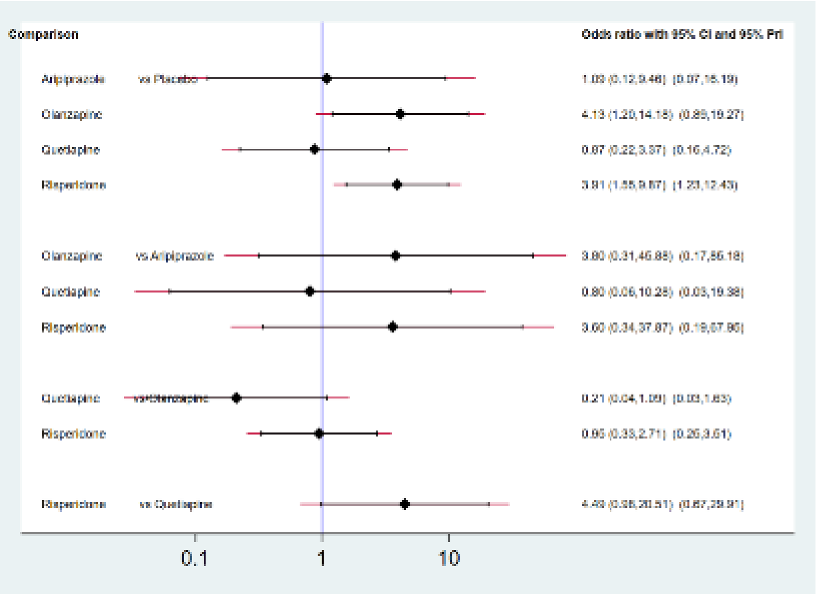

The black solid lines represent the confidence intervals for the Odds Ratios for each comparison and the red dashed lines the respective predictive intervals. The blue line is the line of no effect (ratio equal to 1).

© 2019 Yunusa, I, et al. *JAMA Network Open.*

eReferences

1. Ballard C, Margallo-Lana M, Juszczak E, et al. Quetiapine and rivastigmine and cognitive decline in Alzheimer's disease: randomised double blind placebo controlled trial. *Bmj.* 2005;330(7496):874.
2. Brodaty H, Ames D, Snowdon J, et al. A randomized placebo-controlled trial of risperidone for the treatment of aggression, agitation, and psychosis of dementia. *The Journal of clinical psychiatry.* 2003;64(2):134-143.
3. De Deyn P, Jeste DV, Swanink R, et al. Aripiprazole for the treatment of psychosis in patients with Alzheimer's disease: a randomized, placebo-controlled study. *J Clin Psychopharmacol.* 2005;25(5):463-467.
4. De Deyn PP, Carrasco MM, Deberdt W, et al. Olanzapine versus placebo in the treatment of psychosis with or without associated behavioral disturbances in patients with Alzheimer's disease. *Int J Geriatr Psychiatry.* 2004;19(2):115-126.
5. De Deyn PP, Rabheru K, Rasmussen A, et al. A randomized trial of risperidone, placebo, and haloperidol for behavioral symptoms of dementia. *Neurology.* 1999;53(5):946-955.
6. Deberdt WG, Dysken MW, Rappaport SA, et al. Comparison of olanzapine and risperidone in the treatment of psychosis and associated behavioral disturbances in patients with dementia. *Am J Geriatr Psychiatry.* 2005;13(8):722-730.
7. Katz IR, Jeste DV, Mintzer JE, Clyde C, Napolitano J, Brecher M. Comparison of risperidone and placebo for psychosis and behavioral disturbances associated with dementia: a randomized, double-blind trial. Risperidone Study Group. *The Journal of clinical psychiatry.* 1999;60(2):107-115.
8. Kurlan R, Cummings J, Raman R, Thal L. Quetiapine for agitation or psychosis in patients with dementia and parkinsonism. *Neurology.* 2007;68(17):1356-1363.
9. Paleacu D, Barak Y, Mirecky I, Mazeh D. Quetiapine treatment for behavioural and psychological symptoms of dementia in Alzheimer's disease patients: a 6-week, double-blind, placebo-controlled study. *Int J Geriatr Psychiatry.* 2008;23(4):393-400.
10. Rainer M, Haushofer M, Pfolz H, Struhal C, Wick W. Quetiapine versus risperidone in elderly patients with behavioural and psychological symptoms of dementia: efficacy, safety and cognitive function. *Eur Psychiatry.* 2007;22(6):395-403.
11. Schneider LS, Tariot PN, Dagerman KS, et al. Effectiveness of atypical antipsychotic drugs in patients with Alzheimer's disease. *N Engl J Med.* 2006;355(15):1525-1538.
12. Street JS, Clark WS, Gannon KS, et al. Olanzapine treatment of psychotic and behavioral symptoms in patients with Alzheimer disease in nursing care facilities: a double-blind, randomized, placebo-controlled trial. The HGEU Study Group. *Arch Gen Psychiatry.* 2000;57(10):968-976.
13. Streim JE, Porsteinsson AP, Breder CD, et al. A randomized, double-blind, placebo-controlled study of aripiprazole for the treatment of psychosis in nursing home patients with Alzheimer disease. *Am J Geriatr Psychiatry.* 2008;16(7):537-550.
14. Tariot PN, Schneider L, Katz IR, et al. Quetiapine treatment of psychosis associated with dementia: a double-blind, randomized, placebo-controlled clinical trial. *Am J Geriatr Psychiatry.* 2006;14(9):767-776.
15. Zhong KX, Tariot PN, Mintzer J, Minkwitz MC, Devine NA. Quetiapine to treat agitation in dementia: a randomized, double-blind, placebo-controlled study. *Curr Alzheimer Res.* 2007;4(1):81-93.
16. Mintzer J, Greenspan A, Caers I, et al. Risperidone in the treatment of psychosis of Alzheimer disease: results from a prospective clinical trial. *Am J Geriatr Psychiatry.* 2006;14(3):280-291.
17. Mintzer JE, Tune LE, Breder CD, et al. Aripiprazole for the treatment of psychoses in institutionalized patients with Alzheimer dementia: a multicenter, randomized, double-blind, placebo-controlled assessment of three fixed doses. *Am J Geriatr Psychiatry.* 2007;15(11):918-931.

© 2019 Yunusa, I, et al. *JAMA Network Open.*
